# Genetic insights into dispersal distance and disperser fitness of African lions (*Panthera leo*) from the latitudinal extremes of the Kruger National Park, South Africa

**DOI:** 10.1186/s12863-018-0607-x

**Published:** 2018-04-03

**Authors:** Pim van Hooft, Dewald F. Keet, Diana K. Brebner, Armanda D. S. Bastos

**Affiliations:** 1 Resource Ecology Group, Wageningen University, Wageningen, Netherlands; 2 Department of Zoology & Entomology, Mammal Research Institute,, University of Pretoria, Hatfield, South Africa; 3 Veterinary Services, Kruger National Park, Skukuza, South Africa; 4 Department of Veterinary Tropical Diseases, University of Pretoria, Onderstepoort, South Africa; 5 Phalaborwa, Limpopo Province South Africa

**Keywords:** Lion, *Panthera leo*, Microsatellite, Mitochondrial DNA, RS-3, Kruger National Park, Gene flow, Dispersal, Disease spread, Management

## Abstract

**Background:**

Female lions generally do not disperse far beyond their natal range, while males can disperse distances of over 200 km. However, in bush-like ecosystems dispersal distances less than 25 km are reported. Here, we investigate dispersal in lions sampled from the northern and southern extremes of Kruger National Park, a bush-like ecosystem in South Africa where bovine tuberculosis prevalence ranges from low to high across a north-south gradient.

**Results:**

A total of 109 individuals sampled from 1998 to 2004 were typed using 11 microsatellite markers, and mitochondrial RS-3 gene sequences were generated for 28 of these individuals. Considerable north-south genetic differentiation was observed in both datasets. Dispersal was male-biased and generally further than 25 km, with long-distance male gene flow (75–200 km, detected for two individuals) confirming that male lions can travel large distances, even in bush-like ecosystems. In contrast, females generally did not disperse further than 20 km, with two distinctive RS-3 gene clusters for northern and southern females indicating no or rare long-distance female dispersal. However, dispersal rate for the predominantly non-territorial females from southern Kruger (fraction dispersers ≥0.68) was higher than previously reported. Of relevance was the below-average body condition of dispersers and their low presence in prides, suggesting low fitness.

**Conclusions:**

Large genetic differences between the two sampling localities, and low relatedness among males and high dispersal rates among females in the south, suggestive of unstable territory structure and high pride turnover, have potential implications for spread of diseases and the management of the Kruger lion population.

**Electronic supplementary material:**

The online version of this article (10.1186/s12863-018-0607-x) contains supplementary material, which is available to authorized users.

## Background

The dispersal characteristics of wildlife species, particularly dispersal distance, disperser fitness and body-condition dependency, play an important role in shaping population genetic structure, inbreeding avoidance, and the spatial extent of a population [1–3]. Dispersal is also a critical component in the spread of wildlife diseases [4]. Diseases affect host dispersal through behavioural changes and decreased body condition [4, 5]. In the case of predators, infection may occur through consumption of infected prey [4].

In this study, we conducted a population genetic analysis of the dispersal characteristics of lions (*Panthera leo*) in the Kruger National Park in relation to their fitness. Kruger National Park, a 19,485 km^2^ wildlife reserve, harbours one of the largest lion populations in Africa [6, 7]. The Kruger lion population fluctuates at around 1700 individuals and seems to have remained stable over a period of at least 30 years [8], although it may have decreased somewhat since 2005 [9]. Wildlife diseases, such as foot and mouth disease, anthrax, rift valley fever, brucellosis, feline immunodeficiency virus (FIV) and bovine tuberculosis (BTB) which occur in Kruger, are likely influenced by predator-prey interactions and animal dispersal [8, 10–12]. BTB, which is now an endemic disease in Kruger, particularly among African buffalo (*Syncerus caffer*) [13, 14], was first reported in lions in 1996 [15]. Although BTB-positive lions usually die within a few years, following infection [16], lion numbers do not seem to be significantly affected, at least not yet [8, 11]. FIV is also endemic in the Kruger lion population with FIV-positivity being significantly related to sex (males more likely to be FIV-positive), advanced age and low body condition [11].

The social organisation of lions is well known, consisting of prides of related adult females (average genetic relatedness at half-sib level [17, 18]) and their dependent offspring, together with unrelated adult males. The home range of a pride is stable and may persist for generations [19–21]. Until recently, lions were believed to have a strict within-pride mating system, but recent studies have shown extra-group paternity in Etosha National Park (Namibia) and possibly also Selous Game Reserve (Tanzania) [17, 18]. On average, subadult lions start leaving their natal pride at 29–40 months of age [22–24]. Prides exhibit inbreeding avoidance; mating between related pride members is rare, males tend to leave prides before their daughters start mating and males generally move far away from their natal pride’s home range [18, 19, 22, 23]. Male coalitions can take over a female pride and evict all previous males and females that are too young to conceive [19, 22–24].

Males can disperse large distances, from 120 km in the semi desert of Kgalagadi Transfrontier Park (southern Africa) to more than 200 km in plains-like ecosystems such as Serengeti National Park and Ngorongoro Crater (Tanzania) [22, 25, 26]. In the Serengeti it was shown that 69% of the males left a 2000 km^2^ study area and at least half of the breeding males came from outside this area [19]. Also in the woodland and bushland savannah of Hwange National Park (Zimbabwe) males seem to disperse considerable distances [25]. However, in some bush-like ecosystems such as Selous and Kruger, reported dispersal distances are considerably shorter, namely 20 to 30 km [18, 22, 27]. In Kruger up to 80% of male coalitions in the south-east remained close to their natal territory [22], compared to only 27% of the adult males (7/26) in Kgalagadi [26]. This may result in increased levels of inbreeding in Kruger, even if close inbreeding within prides is avoided [22]. However, it should be noted that the study in Kruger was based on a relatively small region (700 km^2^) in the extreme southeast of the park [22].

Females, if not recruited into the natal pride, generally only leave their natal pride as subadults (≤ 4 years) [19]. They generally do not disperse very far beyond their natal range. In Selous and the Seregenti 15–30% of females settled adjacent to their natal territory, and only 3–6% dispersed farther away [23, 27, 28]. In the Serengeti females did not move more than eight km [28], on average, although some individuals were observed to wander as far as 60 km (but returned afterwards) [23]. No long-distance dispersal among 23 subadult females was observed over a 2.5 year period in Kgalagadi [26]. However, 36% and 61% of the dispersing females in respectively Hwange and the Ngorongoro Crater were observed to disperse beyond the immediate surroundings of their natal pride [23, 24]. Despite the latter two observations dispersal is generally male-biased, because almost all, if not all, males will ultimately leave their natal pride [19, 23, 26, 29].

Animals that disperse probably experience a relatively high mortality [19, 22], particularly subadult males [24], which may to a large extent explain the female-biased adult sex ratio in most lion populations (around 70% females) [22, 30]. Dispersing females have a relatively low chance of reaching eight years of age [19] and relatively low reproductive success [23].

Fine-scale population genetic techniques have been employed to gain insights into the lion populations from Selous [18, 31, 32], Etosha [17] and Hwange [25]. The use of genetic techniques has allowed for estimates of dispersal distances [27], genetic relatedness within prides [17, 18], genetic distances among prides [18], extra-group paternity [17], and long-distance dispersal [25].

Here, we analysed 97 lions from 21 prides and 12 singleton lions in Kruger using 11 microsatellite markers. Additionally, the RS-3 repeat region (± 300 bp) of the mitochondrial DNA (mtDNA) displacement loop (D-loop) region was sequenced for eight male and 20 female lions. This genome region often contains the ‘ACGT’ motif and displays length variation similar to that described for microsatellites [33, 34]. Inheritance of mtDNA is strictly maternal, thereby allowing inferences of female-specific dispersal and gene flow [35].

The two regions sampled in this study are separated by more than 200 km. Prides sampled in the far north all occurred north of the Shingwedzi and Phugwane Rivers, an area that was free of BTB at the time of sampling [36]. Whereas the prides sampled in the south were situated south of the Sabie River, a high BTB prevalence area. The main aim was to obtain estimates of the fraction of dispersing individuals and their dispersal distances. Dispersal was estimated in relation to sex and age. Additionally, we attempted to arrive at a fitness estimate for the dispersing individuals relative to the non-dispersers. We also explored whether there was a difference in dispersal characteristics between the two sampled subpopulations which differed with respect to density, adult sex ratio, territory size (own observations), body condition and BTB prevalence (own observations and [11, 36]). We evaluated four hypotheses: 1) dispersal distances are short for both males and females (< 25 km) in bush-like ecosystems [18, 22, 27], 2) the fraction of dispersing males is small (≤ 20%) [22], 3) the fraction of dispersers increases with age peaking at around 40 months of age; the observed onset of male dispersal in southern Kruger [22], and 4) fitness among dispersers is relatively low; indicated by low body condition and high mortality [19, 22–24].

## Methods

### Description of samples

Lions were captured using call-up stations and immobilized with a combination of tiletamine and zolazepam (Zoletil 100, Virbac), prior to handling. Venous blood samples obtained from the medial saphenous vein as soon as possible after anaesthesia were collected in heparin, EDTA and serum Vacutainer tubes, and kept at ambient temperature until further processing, which always occurred within 8–24 h of sampling. The lions were aged based on the dental attrition guidelines of Smuts et al. 1978 [37]. Body condition score (BCS) was assessed according to predetermined criteria that ranged from 1 (very poor) to 5 (excellent) [11]. Lions were micro-chipped so the animals could be recognized at future captures. Background information on these lions can be found in Additional file 1. FIV and BTB infection status according to the protocols described in [11] were determined in respectively 89 and 42 individuals but no significant associations with genetic background were observed.

Blood samples were collected from 97 lions from 21 prides (47 males and 50 females) and 12 singleton lions (eight males and four females) between June 1998 and October 2004 (sample information in Additional file 2). Fifty-one lions, 38 females and 13 males, were sampled from 13 established territorial prides (T). The other 58 lions, 15 females and 43 males, when observed for the first time, were part of a newly established pride or a nomadic pride (non-territorial, NT), or were singleton nomadic individuals. In the south, seven out of 12 prides were non-territorial compared to only one out of nine in the north.

Forty-five individuals came from the far north of Kruger, north of the Shingwedzi and Phugwane Rivers (22.7–23.2 S, 30.0–31.6 E), except for one pride just south of the Shingwedzi River (two individuals sampled) (map of sampling localities in Additional file 3). Of these 45 individuals one male was caught outside Punda Maria Gate after he had killed 28 head of cattle over a period of weeks. Fifty-six individuals came from southern Kruger, south of the Sabie River (25.1–25.6 S, 31.3–32.1 E). Additionally, eight males were caught in private game reserves that border, but have no fence separation with, southern Kruger: four were from Mthethomusha which borders the south-western side of Kruger and four from Sabi Sand, which is located on the western side of Kruger just north of the Sabie River. The average distance between the northern and southern sampling localities was 267 km, with the two nearest sampling points being 201 km apart and the two furthest sampling points being 308 km apart. Lions between these two areas were not sampled, although they occur throughout the whole of Kruger.

The northern and southern subpopulation differed significantly with respect to density (north: 2.9 individuals/100-km^2^, south: 7.5 individuals/100-km^2^), adult female:male sex ratio (north: 1.75:1, south: 1.06:1), female territory size (north: 300 km^2^, south: 70 km^2^), body condition (percentage of individuals with BCS = 5; north: 77%, south: 44%) and BTB prevalence (only observed in the south at the time of sampling with a prevalence of 72% [11, 36]) (personal observations, Additional file 4). Prides in the north with significantly larger territories divided into two or even three subgroups to forage and were not associating or functioning as a pride unit at all times. The low population density and large territory sizes in the north can be attributed to the relatively low prey availability in this part of Kruger [38]. The blood samples were originally collected for a BTB prevalence study [11] and for that reason were confined to one high-prevalence area in the south and one BTB-free area (at the time of sampling) in the north, with the latter area serving as a control.

#### Genetic analyses

DNA was extracted from the blood samples using standard commercial proteinase K / column capture extraction protocols. A panel of 11 microsatellites, selected from Driscoll et al. 2002 [39], were amplified in three multiplex PCR reactions consisting of the following groups: *FCA075*, *FCA126*, *FCA224* and *FCA247* (group one); *FCA032*, *FCA077*, *FCA094*, *FCA097* and *FCA208* (group two); *FCA205* and *FCA275* (group three). Reactions were performed in an Eppendorf Mastercycler (Gradient 5331) in 5 μl volumes consisting of 50% Multiplex PCR Master Mix (Qiagen), 0.1 μM of each primer and 25–50 ng of genomic DNA. After enzyme activation at 95 °C for 15 min, 30 cycles of denaturation at 94 °C for 30 s, primer-annealing at 57 °C for 90 s and extension at 72 °C for 60 s were performed, ending with a final extension step at 60 °C for 30 min. Following heat denaturation, the PCR products were analysed on an ABI 3100 DNA sequencer (Applied Biosystems) using Genescan-500 Rox (Applied Biosystems) as a size standard. The microsatellite genotype data per individual are provided in Additional file 2.

Additionally, we sequenced the RS-3 repeat region of the mtDNA in 28 randomly chosen individuals (four northern males, four southern males, 12 northern females, eight southern females) using primers CR-R (GGTTGGCGTATCTATAGATA) and KB-F (GGTCCTGACTCAGTCAAATA), designed specifically for this study. PCRs containing ~ 200 ng of template DNA were carried out in a 50 μl volume containing 1× PCR buffer, 2 mM MgCl_2_, 0.2 mM dNTP’s, 5% glycerol, 40 μM of each primer and 2.5 U Ex-Taq polymerase (TaKaRa). Amplification was performed on a Perkin Elmer 9600 Thermal Cycler. PCR thermal cycling conditions comprised two cycles of touchdown PCR consisting of denaturation at 96 °C for 20 s, annealing at 60 °C for 30 s and extension at 72 °C for 40 s, followed by 38 cycles of denaturation at 96 °C for 12 s, annealing at 58 °C for 30 s and extension at 72 °C for 40 s, with a final extension step at 72 °C for 1 min.

PCR products purified with the Roche PCR Template Purification Kit were cycle sequenced using BigDye V3.0 and V3.1 (Applied Biosystems ABI, Foster City, CA) and run on an ABI Prism 3100 automated sequencer. Sequences were aligned to a Serengeti lion from Jae-Heup et al. (2001) which was used as reference sequence in DNAMAN (Lynnon BioSoft), and subsequently verified by eye (sequence not in GenBank but given in full in [40]). Regions that contained any ambiguities or unreadable nucleotides were re-sequenced. All mtDNA sequences generated in this study have been deposited in GenBank under accession numbers: MF401594- MF401621.

#### Statistical analyses

FSTAT 2.9.3.2 was used to estimate mean allelic diversity (*A*), mean observed heterozygosity (*H*_o_) and mean expected heterozygosity (*H*_e_) [41] across loci. Significances of differences in *A* between pairs of groups were estimated with the paired samples *t*-test with each locus constituting a paired observation (conducted in SPSS 22). None of the pairwise differences in *A* differed significantly from normality according the Shapiro-Wilk test (*P* > 0.05). The 95% CIs of *H*_o_ and *H*_e_ were estimated by bootstrapping individuals (1000×) and rescaled by multiplying by the ratio of original to bootstrap mean, performed in Excel [42]. Significance of deviations from Hardy-Weinberg equilibrium (50,000 permutations), significance of population differentiation (50,000 permutations, not assuming random mating within populations), linkage disequilibrium (LD) per region (2200 permutations; number of permutations fixed by the nominal level for multiple tests, here 0.05), and Wright’s *F*-statistics (based on allele identity) including bootstrap 95% CIs were estimated with FSTAT 2.9.3.2. As *F*_*ST*_ tends to underestimate the amount of genetic differentiation between populations when heterozygosity and allelic diversity are high, such as with microsatellites [43], we also applied a standardized measure of *F*_*ST*_, *F*_ST_^*^, which corrects for this underestimation (*F*_ST_^*^ = *F*_*ST*_(1 + *H*_*s*_)/(1-*H*_*s*_), where *H*_*s*_ is the average *H*_e_ per population, [43]). Additionally, gene flow was estimated based on private allele frequencies using Genepop (version 4.2) on the web [44, 45].

We also tested to what extent LD resulted in correlations of *A* and *H*_o_ between locus pairs. In case of *A*, average Pearson *r* was estimated in Excel for random pairs of individuals with randomizations performed per region (40 randomizations, 54 unique pairs per randomization). In case of *H*_o_, the per-locus genotypes, rather than individuals, were randomized per region. The *P*-value of the correlation in *H*_o_ between locus pairs was estimated as the fraction of randomizations (100,000 randomizations) that had a larger *χ*^2^ value than the observed data, with the *χ*^2^ value based on the observed and expected frequencies of double homozygotes, double heterozygotes and homozygote-heterozygote pairs. The expected frequencies, assuming independence of loci, were derived from *H*_o_ at each locus.

Analyses of pairwise relatedness according to Lynch and Ritland (*r*_LR_) [46] were conducted with Genalex 6.4 [47]. Mean and 95% CI of *r*_LR_ (jack-knifed over loci) for groups of individuals were estimated with Spagedi [48]. Mean and 95% CI for *r*_LR_ between groups within prides, i.e. between males and females and between young and old animals, were estimated using the individual pairwise *r*_LR_ values, each with equal weight and under the assumption that they were largely independent from each other (conducted in SPSS 22).

Isolation-by-distance within regions was tested using *r*_LR_ between pairs of individuals as the dependent variable and untransformed geographical distances as the independent variable. We excluded individuals younger than three years because these probably were mostly pre-dispersal (average male age at dispersal in southern Kruger = 40 months [22], see also Fig. 4). Probability of isolation-by-distance was obtained by permutation of observed GPS coordinates among sampling localities, performed in Excel. With this permutation scheme, individuals that have identical GPS coordinates in the observed data set (i.e. from the same pride) also have identical GPS coordinates in the randomized data sets. Probability was estimated as the fraction of random data sets, using 100,000 permutations, showing a Pearson correlation at least as strong as the observed data. Because zero distances were not permutated, they were excluded from the correlations. This procedure amounts to a traditional Mantel test [49]. We choose to perform the Mantel test in Excel in order to be able to analyse genetic distances on basis of *r*_LR_ between individual pairs but at the same time randomize GPS coordinates at the group (pride) level. The fact that samples were collected in different years and that the sampled individuals were of different age did not have a substantial influence on the observed patterns of isolation-by-distance (Additional file 5).

Population assignments were performed with Structure 2.3.4, using the admixture model assuming correlated allele frequencies, with 10 runs per number of clusters (K) for K = 1–6 with 100,000 iterations and a burn-in of 100,000. The assumption of correlated allele frequencies was applied because allele frequencies were strongly correlated between northern and southern Kruger (Pearson *r* = 0.734, *n*_alleles_ = 75). The most supported partitioning (K) was identified using the method of Evanno et al. 2005 [50]. Kendall’s Tau-b (*τ*_*b*_) correlation coefficient and (logistic) regression analysis were used to estimate associations between age, body condition and percentage locally assigned genetic make-up, in SPSS 22.

We did not perform parentage analyses because the non-exclusion probabilities (probability that unrelated individuals were not excluded as possible parents), determined in Cervus 3.0 [51], were too high (0.0167 for northern Kruger and 0.0109 for southern Kruger). We estimated number of haplotypes and haplotype diversity (*D* or gene diversity, which is the probability of randomly sampling two different haplotypes, similar to *H*_e_ for diploid markers) of the RS-3 region with the Excel add-in Microsatellite Toolkit [52]. Minimum spanning networks were constructed that minimized repeat number differences among haplotypes (“by hand” as there were only few mutations).

Exact probabilities of pairwise differences in binomial proportions (Fisher’s exact test) and of χ^2^ tests were calculated with SPSS 22. Error bars in figures that represent 95% confidence intervals of binomial proportions were estimated according to Wilson [53]. Probabilities from different independent tests were combined with the Stouffer’s Z-test [54]. All reported *P*-values are two-sided.

## Results

### Basic microsatellite diversity parameters per region and Wright’s *F*-statistics

There was significant population differentiation between lions occurring in northern and southern Kruger (*P* < 0.00001). Within these two regions and with all samples per region pooled no significant deviations from Hardy-Weinberg equilibrium were observed (*P* = 0.79). Significant LD (Bonferroni corrected α-level: *P* = 0.00046) was observed only between microsatellites *FCA032* and *FCA075*, in both regions. This was probably due to physical linkage as both microsatellites occur on chromosome A2 in the domestic cat (*Felis catus*) [55]. LD did not result in a significant correlation in *H*_o_ between these two microsatellites (*χ*^2^ test: *P* = 0.11) and only a weak correlation in *A* (average Pearson *r* = 0.30).

Wright’s *F*-statistics showed a small genetic distance between northern and southern Kruger. (*F*_*IS*_ = − 0.013, 95% CI: [− 0.056, 0.030]; *F*_*ST*_ = 0.039, 95% CI: [0.023, 0.056]; *F*_*IT*_ = 0.027; 95% CI: [− 0.017, 0.071]). However, the *F*_ST_^*^ value was 0.24 (95% CI: [0.14, 0.35]), which is quite large, indicating only limited gene flow between the two regions. Indeed, some private alleles had quite high frequencies indicative of just 0.62 migrants per generation (two private alleles in the south with frequency > 0.16 and six private alleles in the north with frequency > 0.10). Genetic diversity levels in the two regions were almost identical (northern Kruger: *H*_o_ = 0.731, 95% CI: [0.694, 0.768]; *H*_e_ = 0.722, 95% CI: [0.703, 0.741]; *A* = 5.64; southern Kruger: *H*_o_ = 0.736, 95% CI: [0.705, 0.767]; *H*_e_ = 0.726, 95% CI: [0.711, 0.741]; *A* = 6.02; rarefaction *A* based on a sample size of 45 individuals, no significant difference in *A* between the two regions: *P* = 0.20).

### Genetic relatedness within prides

**Fig. 1:**
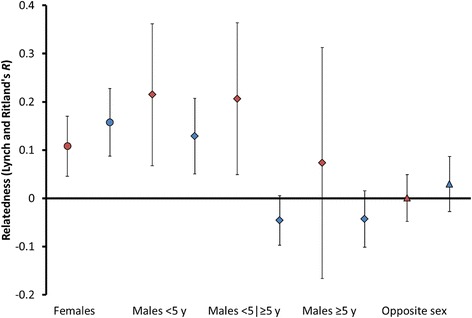
Average pairwise relatedness within prides according to sex and age. Red data points: northern Kruger, blue data points: southern Kruger, circles: females, diamonds: males, triangles: opposite sex, error bars: 95% confidence interval

In both northern and southern Kruger females within prides were significantly related, with average pairwise relatedness being at the level of first cousins (northern Kruger: 0.11, 95% CI: [0.05, 0.17], *n*_*r*-values_ = 42, *n*_individuals_ = 21, *n*_prides_ = 5 T (territorial); southern Kruger: 0.16, 95% CI: [0.09, 0.23], *n*_*r*-values_ = 41, *n*_individuals_ = 26, *n*_prides_ = 3 T-4NT (non-territorial); Fig. 1). The average relatedness of opposite-sex pairs was not significant within prides (i.e. not significantly different from zero), neither in northern Kruger nor in southern Kruger (northern Kruger: 0.00, 95% CI: [− 0.05, 0.05], *n*_*r*-values_ = 27, *n*_individuals_ = 22, *n*_prides_ = 4 T; southern Kruger: 0.03, 95% CI: [− 0.03, 0.09]; *n*_*r*-values_ = 57, *n*_individuals_ = 37, *n*_prides_ = 2 T-3NT; Fig. 1). The low opposite-sex relatedness indicates that males from other prides had entered the focal prides.

In southern Kruger, the average male pair within a pride was not significantly related (0.04, 95% CI: [− 0.01, 0.08]; *n*_*r*-values_ = 49, *n*_individuals_ = 29, *n*_prides_ = 2 T-6NT). However, there was a strong contrast between pairs of males < 5 years and pairs of males that included at least one older male. Only pairs of males < 5 years were on average significantly related (< 5 year: 0.13, 95% CI: [0.05, 0.21], *n*_*r*-values_ = 17, *n*_individuals_ = 15, *n*_prides_ = 4NT; < 5 years vs. ≥ 5 years: -0.05, 95% CI: [− 0.10, 0.01], *n*_*r*-values_ = 16, *n*_individuals_ = 12, *n*_prides_ = 2NT; ≥ 5 years: -0.04, 95% CI: [− 0.10, 0.02], *n*_*r*-values_ = 10, *n*_individuals_ = 14, *n*_prides_ = 2 T-4NT; Fig. 1). Neither males < 5 years nor older males were significantly related to the opposite sex (< 5 years: -0.04, 95% CI: [− 0.12, 0.04], *n*_*r*-values_ = 16, *n*_males_ = 12, *n*_prides_ = 2NT; ≥ 5 years: 0.06, 95% CI: [− 0.02, 0.13], *n*_*r*-values_ = 41, *n*_individuals_ = 30, *n*_prides_ = 2 T-3NT).

In northern Kruger, male pairs within prides were on average significantly related (0.17, 95% CI: [0.09, 0.26], *n*_*r*-values_ = 19, *n*_individuals_ = 17, *n*_prides_ = 5 T-1NT), and significantly more so than male pairs within prides in southern Kruger (*P* < 0.01; Student’s *t-*test using Jacknifed SE estimates). Average male relatedness was significant not only for pairs < 5 years but also for pairs that included one older individual (< 5 year: 0.21, 95% CI: [0.08, 0.35], *n*_*r*-values_ = 6, *n*_individuals_ = 9, *n*_prides_ = 3 T-1NT; < 5 years vs. ≥ 5 years: 0.21, 95% CI: [0.05, 0.36], *n*_*r*-values_ = 8, *n*_individuals_ = 8, *n*_prides_ = 1 T-1NT; ≥ 5 years: 0.07, 95% CI: [− 0.17, 0.37]; *n*_*r*-values_ = 5, *n*_individuals_ = 7, *n*_prides_ = 2 T-1NT; Fig. 1). Males ≥5 years were not significantly related to the opposite sex (≥ 5 years: -0.01, 95% CI: [− 0.06, 0.04], *n*_*r*-values_ = 25, *n*_individuals_ = 20, *n*_prides_ = 4 T; < 5 years: 0.27, no 95% CI because *n*_*r*-values_ = 3, *n*_individuals_ = 5, *n*_prides_ = 2 T). High male relatedness of pairs that included an individual ≥5 years indicates that at least in the two sampled prides from which the data were derived some related males stayed together into adulthood.

### Allelic diversity per pride and Wright’s *F*-statistics at pride level

Average *A* per pride was significantly higher among males than among females, which indicates that males from other prides had entered the focal prides (northern Kruger: *P* = 0.034, southern Kruger: *P* = 0.072; northern and southern Kruger pooled: sex difference = 0.30, 95%: [0.09–0.51], *n*_males_ = 21, *n*_females_ = 25, *n*_prides_ = 3 T-3NT, *P* = 0.0060 (*n* = 66, *n*_loci_*n_prides_); only prides with ≥2 individuals per sex sampled). Genetic distance among prides was similar in both regions with *F*_ST_ being 0.077 in northern Kruger (95% CI: [0.049–0.108]; *n*_individuals_ = 36, *n*_prides_ = 6 T-1NT with ≥3 individuals per pride) and 0.063 in southern Kruger (95% CI: [0.040–0.088]; *n*_individuals_ = 50, *n*_prides_ = 3 T-5NT with ≥3 individuals per pride). These *F*_ST_ values are larger than that observed between northern and southern Kruger, which is probably due the presence of family members. This is supported by a significant negative average *F*_IS_ within prides per region per sex (each of the four combinations: *P* < 0.05; overall average: -0.110, 95% CI: [− 0.159, − 0.060]), which indicates the presence of multiple family members of both sexes per pride (sampling of family members results in a negative bias of *A* and thereby of *H*_*e*_ and *F*_IS_ as well).

### Singleton lions

In both northern and southern Kruger *A* of the pooled singleton lions was similar to that of the pooled pride lions (northern Kruger: singleton lions: *A* = 3.91, pride lions: *A* = 4.01; southern Kruger: singleton lions: *A* = 4.20, pride lions: *A* = 4.18; rarefaction *A* based on a sample size of five individuals, *P* > 0.75). This indicates that the singleton lions originated from a relatively large number of prides. The fraction of individuals > 8 years was relatively large among singleton lions in both northern and southern Kruger (northern Kruger: *P* = 0.013, southern Kruger: *P* = 0.071; pooled samples: *P* = 0.0022, singleton lions: fraction = 0.50, 95% CI: [0.25, 0.75], *n*_individuals_ = 12 (*n*_males_ = 8, *n*_females_ = 4), pride lions: fraction = 0.10, 95% CI: [0.06, 0.18], *n*_individuals_ = 97). Consequently, the fraction of singletons was considerably larger among lions > 8 years than among younger lions (> 8 years: fraction = 0.38, 95% CI: [0.18, 0.61], *n*_individuals_ = 16; ≤ 8 years: fraction = 0.06, 95% CI: [0.03, 0.13], *n*_individuals_ = 93).

### Isolation-by-distance per region

**Fig. 2:**
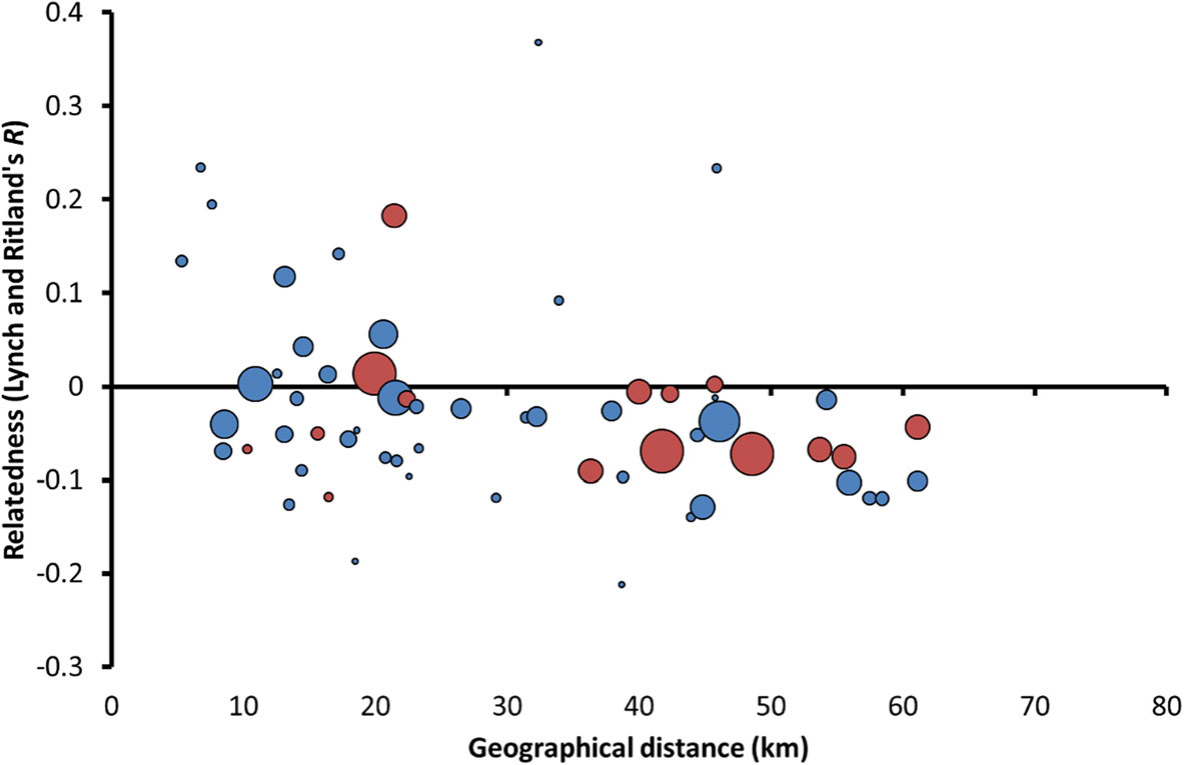
Isolation-by-distance among females ≥3 year old. Red circles: northern Kruger, blue circles: southern Kruger. Circles represent mean relatedness per pair of localities and circle size total number of comparisons per pair of localities (minimum = 1, maximum = 36). Northern Kruger: Pearson *r* = − 0.22, *P* = 0.039, *n*_individuals_ = 23, *n*_localities_ = 6; southern Kruger: Pearson *r* = − 0.23, *P* = 0.014, *n*_individuals_ = 26, *n*_localities_ = 10

There was significant isolation-by-distance among females ≥3 years but not among males ≥3 years (northern females: Pearson *r* = − 0.22, *P* = 0.039, *n*_individuals_ = 23, *n*_localities_ = 6; southern females: Pearson *r* = − 0.23, *P* = 0.014, *n*_individuals_ = 26, *n*_localities_ = 10; northern males: Pearson *r* = − 0.08, *P* = 0.30, *n*_individuals_ = 19, *n*_localities_ = 9; southern males: Pearson *r* = − 0.03, *P* = 0.32, *n*_individuals_ = 28, *n*_localities_ = 9; Fig. 2 and Additional file 6). Absence of significant isolation-by-distance among males is indicative of male-biased dispersal at a geographical scale smaller than 75 km. Females from different prides were related (average *r*_LR_ between pairs of prides > 0) up until a distance of around 20 km, indicating that most females did not disperse beyond this distance.

### Microsatellite cluster analysis: Three clusters

**Fig. 3:**
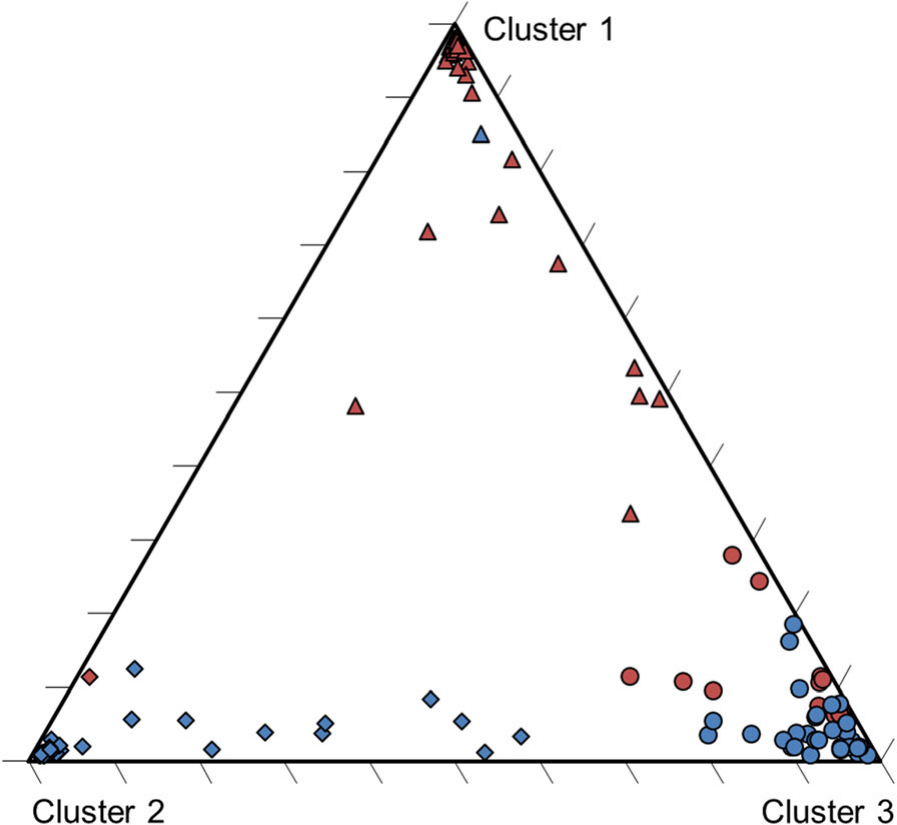
Triangle plot denoting the three microsatellite clusters identified by Structure. Red data points: northern Kruger, blue data points: southern Kruger. The ancestry of each individual was distributed across three clusters by the program Structure. Three groups of individuals could be identified. Triangles: group 1 with mostly individuals from northern Kruger (northern cluster group); diamonds: group 2 with mostly individuals from southern Kruger (southern cluster group); circles: group 3 with individuals from both regions (mixed cluster group)

Three microsatellite clusters were identified by Structure (Fig. 3 and Additional file 7). Clusters 1 and 2 were typical for respectively northern Kruger and southern Kruger, while cluster 3 could not be assigned to a specific region. Three groups of individuals could be identified: group 1 with at least 33% of the genetic make-up of each individual assigned to cluster 1 (northern cluster group), group 2 with at least 41% of the genetic make-up of each individual assigned to cluster 2 (southern cluster group), group 3 with at least 61% of the genetic make-up of each individual assigned to cluster 3 (mixed cluster group). Genetic drift among closely related females and young males (< 5 years) within prides probably resulted in the development of one local cluster per region, with the third cluster (mixed cluster group) being due to dispersers that originated from unsampled prides in the two study areas and the neighbouring areas south of the Shingwedzi River and north of the Sabie River.

The mixed-group individuals from northern and southern Kruger differed from each other by an *F*_*ST*_ value significantly larger than zero (*F*_*ST*_ = 0.024, 95% CI: [0.010, 0.041]; *F*_ST_^*^ = 0.15, 95% CI: [0.06, 0.25]), which can therefore be considered as belonging to two distinct cluster subgroups. The northern and southern cluster group were differentiated by a considerably larger *F*_ST_ value (*F*_*ST*_ = 0.080, 95% CI: [0.046, 0.118]; *F*_ST_^*^ = 0.45, 95% CI: [0.26, 0.66]). Two candidate dispersers or offspring thereof were identified: one eight year old male from the northern cluster group sampled in the south and one seven year old female from the southern cluster group sampled in the north. The eight year old male was originally identified for the first time in a nomadic group.

*A* was significantly lower in the regional cluster groups than in the mixed cluster subgroups (northern cluster group: 4.44, northern mixed cluster subgroup: 5.09, *P* = 0.052; southern cluster group: 4.68, southern mixed cluster subgroup: 5.13, *P* = 0.10; difference after pooling the northern and southern samples = 0.55, 95% CI: [0.15, 0.95], *P* = 0.0089 (*n* = 22, *n*_loci_*n_regions_), based on sample size of 12 individuals). This difference in *A* was still seen when in the regional cluster groups only one individual per pride was included and all singleton lions were excluded (difference = 0.38, 95% CI: [− 0.03, 0.79], *P* = 0.065, rarefaction *A* based on sample size of seven individuals). More specifically, from each pride the one with the highest probability of being pre-dispersal was selected by choosing the youngest individual, preferentially a female. The latter result indicates that the relatively low *A* in the regional cluster groups is unlikely to be attributed to the inclusion of multiple members from the same family or pride. High *A* in the two mixed cluster subgroups and the relatively small genetic distance between them indicates that these included relatively many dispersers (i.e. outside their natal pride) that originated from a large number of unsampled prides.

*A* was higher among the males than among the females in the mixed cluster subgroups, particularly in northern Kruger (northern male subgroup: 4.26, northern female subgroup: 3.73, *P* = 0.013; southern male subgroup: 4.11, southern female subgroup: 4.04, *P* = 0.74; difference after pooling the northern and southern samples = 0.30, 95% CI: [0.01, 0.59], *P* = 0.045 (*n* = 22, *n*_loci_*n_regions_), rarefaction *A* based on a sample size of five individuals). No significant sex difference was observed in the regional cluster groups (northern Kruger: *P* = 0.25, southern Kruger *P* = 0.061 (opposite sign); pooled samples: *P* = 0.33, based on sample size of five individuals). A relatively high *A* among males in the mixed cluster subgroups indicates that they originated from a larger number of prides than the females and that they thus had travelled relatively large distances.

### Microsatellite cluster analysis: Candidate local dispersers and residents

The genetic make-up of individual lions in both northern and southern Kruger, as derived from the inferred cluster proportions per individual, showed a bimodal distribution (Additional files 8 and 9). The highest frequency of individuals (87 out of 109) had either less than 20% (northern Kruger: 0.24, 95% CI: [0.14, 0.39]; southern Kruger: 0.58, 95% CI: [0.46, 0.69]) or more than 90% (northern Kruger: 0.51, 95% CI: [0.37, 0.65]; southern Kruger: 0.25, 95% CI: [0.16, 0.37]) of their genetic make-up assigned to one of the local clusters (i.e. cluster 1 and 2). Most of the former probably were dispersers (i.e. outside their natal pride; candidate dispersers), and most of the latter residents (i.e. inside their natal pride; candidate residents), indicating that northern Kruger was characterized by a relatively small fraction of dispersers (0.24 vs 0.58 in southern Kruger) and high fraction of residents (0.51 vs. 0.25 in southern Kruger) (Additional file 8). Relatively many individuals (22 out of 109) with between 20% and 90% of their genetic make-up assigned locally probably had both dispersers and residents in their recent ancestry (mixed ancestry), with a higher percentage indicating a more recent local ancestry. In the south, these were mostly female (*χ*^2^ = 6.55, *P* = 0.047; observed: 9 out of 11, expected: 5.2 out of 11; no significant sex difference in the north: *χ*^2^ = 1.28, *P* = 0.63). The designation of candidate disperser status to an individual based on microsatellite cluster analysis was supported by a high fraction of candidate dispersers among males compared to territorial females (*P* = 0.019, see next section).

### Microsatellite cluster analysis: Disperser status associated with age and body condition

**Fig. 4:**
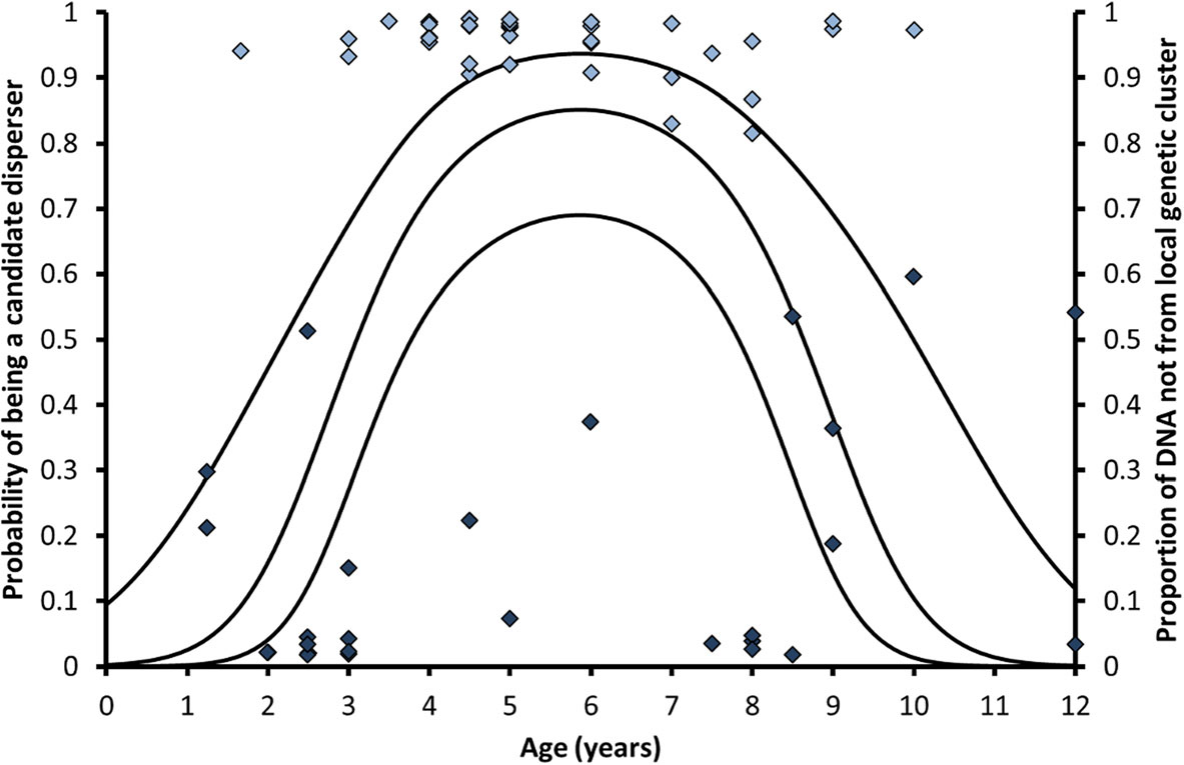
Relationship between age and dispersal in southern Kruger (logistic regression). Regression line: probability of being a candidate disperser. Upper and lower lines: upper and lower limit of the 95% confidence interval of the regression line. Data points: proportion of DNA not coming from the local cluster (cluster 2, right axis). Light blue diamonds: > 80% of DNA not coming from the local cluster and therefore considered as candidate dispersers; dark blue diamonds: > 40% of DNA coming from the local cluster therefore considered as candidate residents. Dependent variable: candidate disperser status (value = 1: candidate disperser, proportion of DNA that was not assigned to the local cluster > 80%, *n*_individuals_ = 37; value = 0: candidate resident, *n*_individuals_ = 27), *χ*2-value model = 20.5, d.f. = 2, *P*_model_ = 0.000036, *P*_age_ = 0.00058, *P*_age2_ = 0.00070. Sex was not significant when added to the regression model (*P*_sex_ = 0.78)

In southern Kruger there was a hump-shaped relationship between the inferred proportion of disperser ancestry (i.e. cluster 1 and 3) and age (adj. *R*^2^ = 0.17, *P*_model_ = 0.0015, *P*_age_ = 0.00033, *P*_age2_ = 0.00040; right axis Fig. 4). Individuals of intermediate age had the highest proportion of disperser ancestry. A second-order logistic regression, whereby individuals with less than 20% local ancestry were considered as dispersers or offspring thereof, better described the relationship between disperser status and age (*χ*^2^ = 20.5, *P*_model_ = 0.000036, *P*_age_ = 0.00058, *P*_age2_ = 0.00070; Fig. 4). Sex was not significant when included in the regression model (*P*_sex_ = 0.78). The regression was significant for both sexes (males: *P*_model_ = 0.050, females: *P*_model_ = 0.0011). Differences in the fraction of candidate dispersers were especially stark when comparing the age classes 1–3 years, 3.5–7 years and > 7 years. As many as 90% (95% CI: [73%, 98%], *n*_individuals_ = 30) of the 3.5–7 year old individuals were candidate dispersers, in contrast to just 29% (95% CI: [17%, 46%], *n*_individuals_ = 34) of the individuals in the other two age classes. The percentage candidate dispersers among females of 3.5–7 years old was at least 68% (*P* = 0.05, *n*_individuals_ = 11). Most of the females in this age group were non-territorial (82%, 95% CI: [52, 95%], *n*_individuals_ = 11).

No significant logistic regression or differences among the three earlier mentioned age classes were observed in northern Kruger (*P >* 0.30). As with southern Kruger, there was no obvious sex-biased dispersal (male fraction among candidate dispersers: 0.64, 95% CI: [0.35, 0.85], *n*_individuals_ = 11; male fraction among the other individuals: 0.44, 95% CI: [0.29, 0.61], *n*_individuals_ = 35; *P* = 0.31). When pooling northern and southern Kruger, the fraction of candidate dispersers was larger among males than among females, but not significantly so (males: 0.52, 95% CI: [0.39, 0.64], *n*_individuals_ = 56; females: 0.36, 95% CI: [0.24, 0.49], *n*_individuals_ = 53; *P* = 0.12). However, when only the territorial females were included, the fraction of male dispersers was not only two-fold higher, but significantly so (fraction dispersers among territorial females: 0.26, 95% CI: [0.15, 0.42], *n*_individuals_ = 38; *P* = 0.019). In southern Kruger, the fraction of candidate dispersers among non-territorial females (only one non-territorial female was sampled in the north) was as high as that among males (non-territorial females: 0.64, 95% CI: [0.39, 0.84], *n*_individuals_ = 11).

**Fig. 5:**
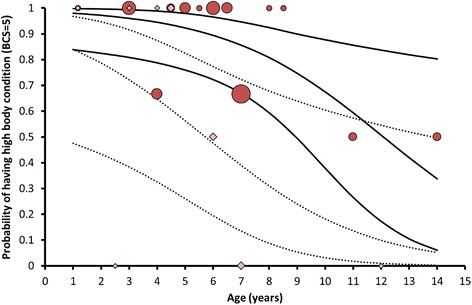
Relationship between age and body condition for residents and dispersers in northern Kruger (logistic regression). Red circles: candidate residents, pink diamonds: candidate dispersers. Size of data points is proportional to the number of sampled individuals (candidate residents: 1–8 individuals, candidate dispersers: 1–2 individuals). Regression lines: probability of having a high body condition. Upper and lower lines: upper and lower limit of the 95% confidence interval of the regression lines. Dotted lines: candidate dispersers, solid lines: candidate residents. Dependent variable: body condition (BCS): 0: BCS ≤ 4 (only two out of 44 individuals with BCS < 4), 1: BCS = 5. *χ*2-value model = 10.71, d.f. = 2, *P*_model_ = 0.0047, *P*_age_ = 0.018, *P*_disperser status_ = 0.018. Sex was not significant when added to the regression model (*P*_sex_ = 0.34). Candidate resident with body condition = 0: *n*_individuals_ = 5, candidate resident with body condition = 1: *n*_individuals_ = 28, candidate disperser with body condition = 0: *n*_individuals_ = 5, candidate disperser with body condition = 1: *n*_individuals_ = 6

According to logistic regression, the probability of having a high body condition (BCS = 5) decreased with age and was relatively low among candidate dispersers in northern Kruger (*χ*^2^ = 10.71, *P*_model_ = 0.0047, *P*_age_ = 0.018, *P*_disperser status_ = 0.018; Fig. 5). In southern Kruger there was a hump-shape relationship between the probability of having a high body condition (BCS = 5) and age, similar to what has been observed for the relationship between the probability of being a candidate disperser and age (*χ*^2^ = 26.83, *P*_model_ < 0.0001, *P*_age_ = 0.00031, *P*_age_^2^ = 0.00025; Additional file 10). However, whether or not an individual was a candidate disperser did not have a significant effect in the model (*P*_disperser status_ = 0.95).

#### MtDNA RS-3 analysis

**Fig. 6:**
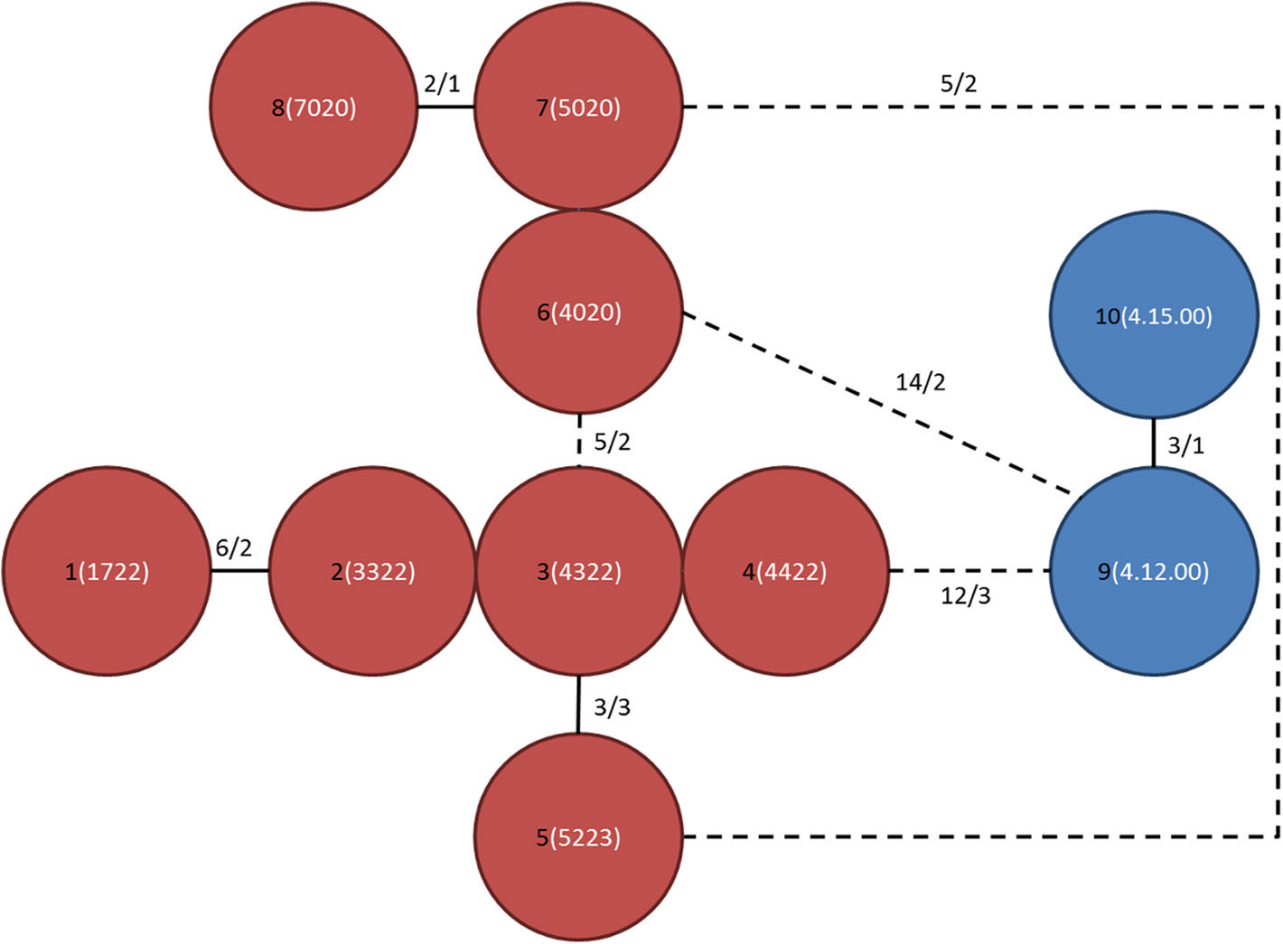
Minimum spanning network of RS-3 sequences of the northern haplotype cluster Red haplotypes: northern Kruger, blue haplotypes: southern Kruger (Sabi Sands). Black numbers: haplotype number, white numbers between brackets: number of repeats per polymorphic site (same order as in Table 1). First site: number of ba-repeats, second site: number of a-repeats, third and fourth site: number of c-repeats. Lines in between haplotypes: total number of repeat differences/total number of polymorphic sites. Dashed lines: alternative connections. No line in between adjoining haplotype indicates difference of one repeat at one polymorphic site. Due to a very different repeat structure, haplotypes from the northern and southern cluster could not be aligned with each other

**Fig. 7:**
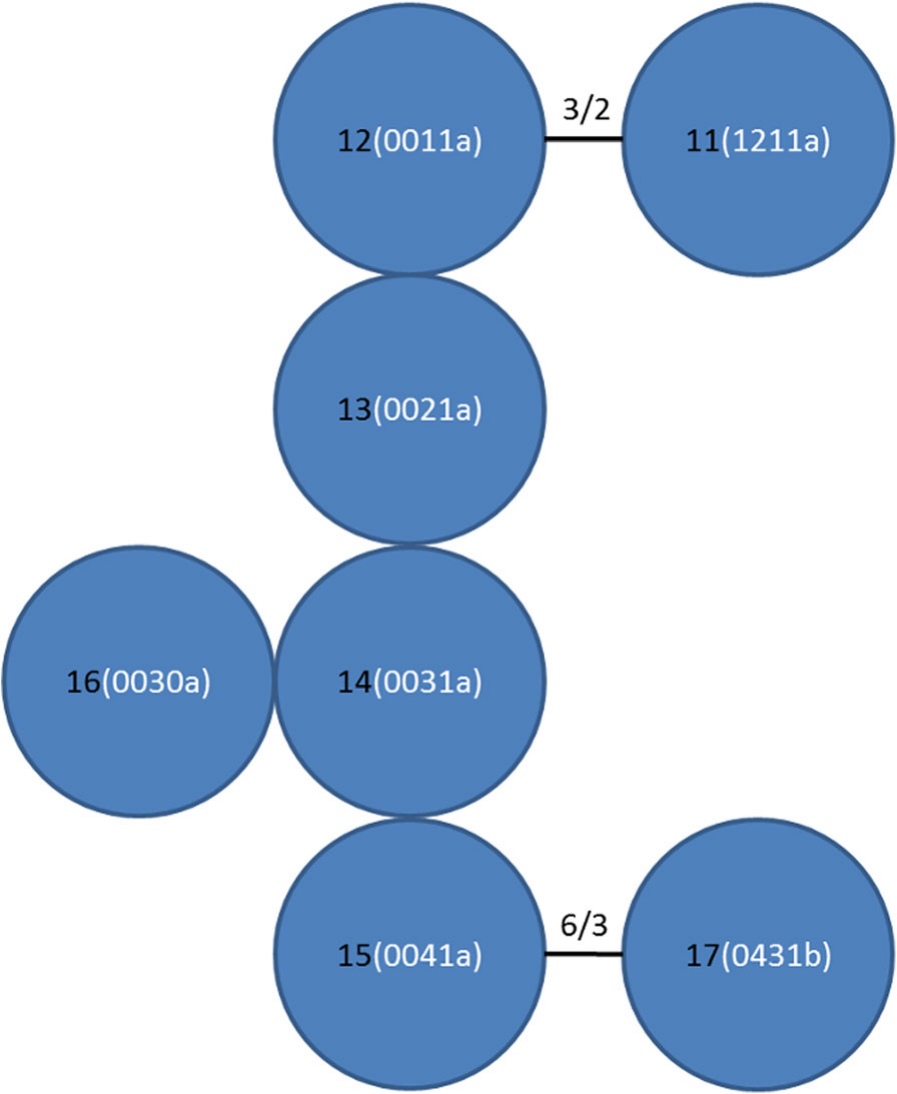
Minimum spanning network of RS-3 sequences of the southern haplotype cluster Black numbers: haplotype number, white numbers between brackets: number of repeats per polymorphic site (same order as in Table 1). First site: number of b-repeats, second and third site: number of a-repeats, fourth site: number of b-repeats, fifth site: a- or b-repeat. Lines in between haplotypes: total number of repeat differences/total number of polymorphic sites. No line in between adjoining haplotype indicates difference of one repeat at one polymorphic site. Due to a very different repeat structure, haplotypes from the northern and southern cluster could not be aligned with each other

Seventeen haplotypes were observed among 28 individuals resulting in a high haplotype diversity (*D* = 0.966). The haplotypes formed two clusters with a very different repeat structure (Table 1) that could not be aligned; one almost exclusively for northern Kruger (except for two young males; northern haplotype cluster) and one specific for southern Kruger (southern haplotype cluster). Four repeat motifs were observed, TACACG (a), TACACACG (b), TATACACG (c) and TACGCACG (d). Within each cluster the haplotypes mainly differed by the number of a-repeats and b-repeats. The minimum spanning networks of these clusters indicated relatively few mutations (Figs. 6 and 7).

**Table 1:** MtDNA haplotypes, encoded repeats of the RS-3 region

					DNA sequence
Reference (Serengeti)					efb|ba2|ba2|ba2|ba5|c-|a8|a13|-|-|-|cg
Northern Kruger					
Ind nr	Sex	Age yr	Social group	H nr	
1	M	3	I	1	efb|ba2|ba2|ba2| ba |cd|a8| a7 |b2| c2 | c2 |cg
2	F	3	K&F	1	efb|ba2|ba2|ba2| ba |cd|a8| a7 |b2| c2 | c2 |cg
3	F^1^	7	G	2	efb|ba2|ba2|ba2| ba3 |cd|a8| a3 |b2| c2 | c2 |cg
4	F	6	C	3	efb|ba2|ba2|ba2| ba4 |cd|a8| a3 |b2| c2 | c2 |cg
5	F	6	K&F	3	efb|ba2|ba2|ba2| ba4 |cd|a8| a3 |b2| c2 | c2 |cg
6	F	4	K&F	3	efb|ba2|ba2|ba2| ba4 |cd|a8| a3 |b2| c2 | c2 |cg
7	F	5	K&F	4	efb|ba2|ba2|ba2| ba4 |cd|a8| a4 |b2| c2 | c2 |cg
8	F	7	G	4	efb|ba2|ba2|ba2| ba4 |cd|a8| a4 |b2| c2 | c2 |cg
9	F	14	G	4	efb|ba2|ba2|ba2| ba4 |c?|a8| a4 |b2| c2 | c2 |cg
10	F	4	Q	5	efb|ba2|ba2|ba2| ba4?a |cd|a8| a2 |b2| c2 | c3 |cg
11	M	1.2	R	6	efb|ba2|ba2|ba2| ba4 |cd|a8| - |b2| c2 | - |cg
12	M	6	K&F	6	efb|ba2|ba2|ba2| ba4 |cd|a8| - |b2| c2 | - |cg
13	F	4	Q	7	efb|ba2|ba2|ba2| ba5 |cd|a8| - |b2| c2 | - |cg
14	F	6	Q	7	efb|ba2|ba?a|ba2| ba5 |??|a8| - |b2| c2 | - |cg
15	M	7	C	8	efb|ba2|ba2|ba2| ba7 |cd|a8| - |b2| c2 | - |cg
16	F	8	Q	8	efb|ba2|ba2|ba2| ba7 |cd|a8| - |b2| c2 | - |cg
Southern Kruger					
Ind nr	Sex	Age yr	Social group	H nr	
Sabi Sands					
17	M	2.5	Hb^#^	9	efb|ba2|ba2|ba2| ba4 |cd|a8| a12 |-| - | - |cg
18	M	2	Hb^#^	10	efb|ba2|ba2|ba2| ba4 |cd|a8| a15 |-| - | - |cg
South of Sabie River					
19	M	4.5	J^#^	11	efb|ba2| ba2 |-aba| a2 |b3|?|a2| a | b | ba |a5|b|a2|b|a3|b3|a|cg
20	F	3	EX-A	12	efb|ba2| -aba |-aba| - |b3|b|a2| a | b | ba |a5|b|a2|b|a3|b3|a|cg
21	F	10	B	12	efb|ba2| -aba |-aba| - |b3|b|a2| a | b | ba |a5|b|a2|b|a3|b3|a|cg
22	F	5	EX-A	13	efb|ba2| -aba |-aba| - |b3|b|a2| a2 | b | ba |a5|b|a2|b|a3|b3|a|cg
23	F	2.5	EX-A	14	efb|ba2| -aba |-aba| - |b3|b|a2| a3 | b | ba |a5|b|a2|b|a3|b3|a|cg
24	M	6	Single^#^	14	efb|ba2| -aba |-aba| - |b3|b|a2| a3 | ? | ba |a5|b|a2|b|a3|b3|a|cg
25	F	12	D	15	efb|ba2| -aba |-aba| - |b3|b|a2| a4 | b | ba |a5|b|a2|b|a3|b3|a|cg
26	F	3	B	16	efb|ba2| -aba |-aba| - |b3|b|a2| a3 | - | ba |a5|b|a2|b|a3|b3|a|cg
27	F	3	B	16	efb|ba2| -aba |-aba| - |b3|b|a2| a3 | - | ba |a5|b|a2|b|a3|b3|a|cg
28	F	10	Single^#^	17	efb|ba2| -aba |-aba| a4 |b3|b|a2| a3 | b | b2 |a5|b|a2|b|a3|b3|a|cg

A = TACACG; b = TACACACG; c = TATACACG; d = TACGCACG, e = TATACGCG, f = CACATGTG, g = TATACACATG. Numbers indicate number of repeats, eg. ba2 = baba. 1: one parent was probably a long-distance disperser according to Structure analysis, locally polymorphic sites are underlined, ?: missing data, −: gap, #: non-territorial individual. Reference sequence: Ple181–1 in [40]

All females genotyped for RS-3 had local haplotypes (i.e. northern females had haplotypes from the northern haplotype cluster and southern females from the southern haplotype cluster) indicating no or only limited female gene flow between northern and southern Kruger. However, structure analysis indicated that one seven year old northern female genotyped for RS-3 was a long-distance disperser or offspring thereof (see section “Microsatellite cluster analysis: three clusters”). Further, two young males (2 and 2.5 years old) sampled in southern Kruger, which were local residents according to microsatellite cluster analysis, had a northern haplotype. Within each social group multiple haplotypes were observed among females (17 females in five territorial groups), indicating local female gene flow among prides.

## Discussion

### Dispersal distances

Various observations point towards male-biased dispersal: 1) high relatedness among females but low opposite-sex relatedness within prides, 2) high *A* among male pride members, 3) large fraction of candidate dispersers among males, 4) no isolation-by-distance among males ≥3 years at distances < 75 km, 5) high *A* among males in the mixed cluster subgroups. Taken together these observations reject the hypothesis of short male dispersal distances (< 25 km) [18, 22, 27]. In contrast, females ≥3 years from different prides were related up until a distance of around 20 km, indicating that most females, at least the reproductively successful ones, did not disperse further away than this distance from their natal pride.

Further, many of the females that settled in a new pride were able to produce offspring as females from the same pride had different mtDNA haplotypes. However, the relatively large fraction of candidate dispersers among females, particularly in southern Kruger, together with the observation of multiple haplotypes among female pride members, indicates that some females were dispersing beyond their natal range. However, the average distance travelled by these females was still shorter than that of the average male considering the relatively low allelic diversity among females assigned to the mixed cluster subgroups.

The genetic make-up (microsatellite data) of two individuals indicated long-distance male gene flow (75–200 km, with 75 km being the maximum distance between samples from a single region and 200 km the minimum distance between samples from northern and southern Kruger), either by themselves (the eight year old male from a nomadic group) or by their father (the seven year old female with a local mitochondrial haplotype). The rate of long-distance male gene flow was at least 0.6% (2 out of 109 samples, *P* = 0.05), while the presence of two haplotype clusters indicated no or only limited long-distance female gene flow. Apparently also in bush-like ecosystems, such as in Kruger, male lions can travel large distances and be reproductively successful, although the elongated shape of Kruger (350 km long but only about 60 km wide) may have played a role in potential dispersing opportunities. However, the relatively large *F*_ST_^*^ value between northern and southern Kruger, the low estimate of the number migrants per generation and the presence of three microsatellite clusters indicate that male long-distance dispersers constitute only a small fraction or have low reproductive success. The north-south comparison only allowed us to study long-distance dispersal and gene flow due to the absence of samples from central Kruger, although their inclusion could have revealed a pattern of gradual isolation-by-distance across the whole of Kruger for both microsatellites and mtDNA.

The two young southern males (2 and 2.5 years old) with a northern mitochondrial haplotype were probably local residents considering their young age, which was supported by microsatellite cluster analysis. Furthermore, their haplotypes deviated from the other northern haplotypes in that they were the only ones without a b2|c2-repeat motif and with the most a-repeat motifs (12–15 vs. 2–7), indicating that these males did not come from the extreme north but from a more southern locality. They were caught at Sabi Sands, the only sampling locality in the south that was north of the Sabie River and it is quite well possible that their haplotypes were typical for Sabi Sands.

### Fraction of dispersers

The hypothesis of a small fraction of dispersing males (≤ 0.2) [22] was confirmed for northern Kruger by two observations: a significant relatedness for male pairs that included one individual ≥5 years, which is indicative of male coalitions staying together into adulthood, and a relatively small fraction of candidate dispersers. In contrast, in southern Kruger male coalitions seemed to break apart before adulthood (no significant relatedness for male pairs that included one individual ≥5 years) and the fraction of candidate dispersers was very large. In northern Kruger *A* was relatively high among males in the mixed cluster subgroup indicating that males travelled larger distances than females. This was supported by the absence of significant isolation-by-distance among males ≥3 years. The combination of small disperser fraction and large dispersal distance among males in northern Kruger may be related to the large territory sizes (due to low prey availability [38]) in this part of the park (on average around 300 km^2^ compared to around 70 km^2^ in southern Kruger, Additional file 4).

The results for southern Kruger with respect to male dispersal are in contradiction to those found in an ecological study in the same region that recorded a low dispersal rate (20%) and small dispersal distances (< 20 km) [22]. One possible explanation for these contradicting results is the small number of male coalitions analysed in that study, together with a lack of identity verification. Twenty percent (2/10, 95% CI: [6%, 51%]) of the sub-adult male coalitions were not seen again after marking, which may have been individuals that had dispersed > 20 km [22]. Our results also contradict the short male dispersal distances reported for another bush-like ecosystem, namely Selous in Tanzania, also based on genetic data (no more than three home ranges apart, which is less than 30 km) [18, 27]. However, in that study system significant isolation-by-distance, which indicates relatively short dispersal distances, was only reported for females and only 14 males were sampled [27]. Furthermore, the estimate of male dispersal is probably inaccurate because it was not directly assessed but indirectly derived from the pattern of isolation-by-distance among females [27]. Finally, numerous relatedness values between 0.1 and 0.2 were observed between individuals ±50–70 km apart (≈ five to seven prides apart) [18], which in Kruger was quite rare, even among males. This indicates that at a considerable fraction of lions in Selous dispersed relatively large distances.

The estimated fraction of female dispersers was similar to that normally reported for lions when only the territorial females were considered (this study: 95% CI: [0.15, 0.42]; 0.15–0.36 in Serengeti, Selous and Hwange [23, 24, 27, 28]). The fraction of candidate dispersers among non-territorial females in southern Kruger was considerably larger (0.64, 95% CI: [0.39, 0.84]), although, as previously mentioned, it is unlikely that most of them travelled more than 20 km. However, also in the densely populated Ngorongoro Crater and in large prides in the Serengeti female dispersal rates > 0.60 have been observed [23, 28].

Not only the high female dispersal rate but also the relatively unstable male coalition structure in southern Kruger (no significant relatedness among coalition member pairs with one individual ≥5 years) may explain the non-territorial status of many of the sampled prides. This was supported by observations of the total eviction of two entire territorial prides (EX-A and Nhlanganzwane, the latter not included in this study) from their territory during the study period (which thereby became non-territorial). The unstable territory structure may be related to the relatively low body condition of lions in southern Kruger. Low body condition may have resulted in less stable male coalitions and high pride turnover which subsequently may have increased female dispersal rate. The low body condition in the south may possibly be associated with the high BTB prevalence in this region, although it should be noted that a recent study failed to find a significant association between BTB infection and body condition [11]. An alternative explanation for the unstable territory structure may be the high lion density in the south, which is a consequence of a high prey biomass [38]. The relative difficulty in defending a territory at high population density may have resulted in more intraspecific aggression [56].

### Dispersal in relation to age and disperser fitness

The hypothesis of an increase in the fraction of male dispersers with age, particularly at 40 months of age [22], was supported by a small fraction of candidate dispersers among 1–3 year old individuals in southern Kruger. The absence of significance in northern Kruger may be due to a small number of 1–3 year old individuals (*n*_individuals_ = 9) in combination with a small number of candidate dispersers (*n*_individuals_ = 11).

The observation of relatively strong population genetic structure (occurrence of three microsatellite clusters, large *F*_ST_^*^ value between north and south, low estimated number of migrants per generation) despite the absence of significant isolation-by-distance among males at distances up to 75 km suggests that male dispersers have relatively low reproductive success. The hypothesis of a relatively low fitness among dispersers [19, 22] was further supported by a relatively low body condition among candidate dispersers in northern Kruger and the probability of disperser candidacy being negatively correlated with old age (> 6 years) in southern Kruger, which may indicate increased mortality. Further, a recent study that included samples from this study revealed that low body condition was associated with an increased probability of being FIV-positive [11]. However, the negative correlation between probability of being a candidate disperser and age may also be attributed to old males being evicted from their previous territorial pride and forced to live as singletons, of which relatively few were sampled (12 out of 109). This would be consistent with the observation that evicted males rarely gain access to a new pride [22]. Further, it has been observed in the Serengeti that males that approach old age tend to abandon one of their prides without taking over a new one while males that abandoned one pride for another were relatively young and vigorous [19]. Old males being evicted from their previous territorial prides was further supported by the relatively old age of the singleton lions included in this study. Emigration by adult females is supposedly relatively rare, but they may occasionally leave the pride with subadults or become separated from the rest when prides divide [19]. This was supported by the relatively small fraction of females among non-territorial individuals observed in this study (non-territorial: 15 out of 58, territorial: 39 out of 51). Whether due to mortality, eviction or both, the decrease in the probability of being a disperser at higher ages indicates a relatively low fitness among dispersers in southern Kruger.

## Conclusions

Genetic differences between the two distant localities of Kruger were quite large, despite the observation of (occasional) long-distance male gene flow, with the genetic structure in the south pointing towards an unstable territory structure and high pride turnover, at least in the years 1998–2004. The differences were so pronounced in the mtDNA that one may even speak of two distinct matrilineal subpopulations, although their divergence may be characterized by isolation-by-distance in the intervening area. We have no reason to suspect the situation is any different today, and these genetic insights could be taken into account in management policies.

The high local dispersal rates, for both males and females, and the observation of long-distance dispersal among males indicate that infectious diseases have the potential to spread rapidly through the population. Considering that dispersers tend to have relatively low body condition, making them more vulnerable to FIV and BTB, and probably other infections [5, 11], exacerbates the disease implications. The relatively unstable male coalitions and high female dispersal rate in the south suggest that causality possibly goes both ways with infections leading to low body condition and to the inability to retain the position in a pride.

## Additional files

Additional file 1: Background information on the analysed lions. (DOCX 25 kb)

Additional file 2: Comma delimited sheet (*.csv) with raw data per individual, including data on microsatellite genotype, mtDNA haplotype number, sampling date, GPS coordinates, age, sex, BCS, FIV status, BTB status, socialisation, territory size, cluster assignment according to Structure. (CSV 29 kb)

Additional file 3: Map of sampling localities. (DOCX 793 kb)

Additional file 4: Table with some characteristics of the northern and southern subpopulation. (DOCX 20 kb)

Additional file 5: No discernible effect of lion age and sampling date on isolation-by-distance analyses. (DOCX 12 kb)

Additional file 6: Figure showing absence of isolation-by-distance among males ≥3 year old. (DOCX 98 kb)

Additional file 7: Figure showing the number of microsatellite clusters based on the software Structure using the method of Evanno et al... 2005. (DOCX 52 kb)

Additional file 8: Figure showing the fraction of candidate residents, candidate dispersers and individuals with mixed ancestry per locality. (DOCX 37 kb)

Additional file 9: Figure showing the cumulative frequency distribution of the proportion of DNA per individual assigned to the local microsatellite cluster by the software Structure. (DOCX 56 kb)

Additional file 10: Figure showing the relationship between body condition and age per locality (logistic regression). (DOCX 72 kb)

## Abbreviations

BCS: Body condition score
BTB: Bovine tuberculosis
FIV: Feline immunodeficiency virus
LD: Linkage disequilibrium
NT: Non-territorial
T: Territorial
