# Supplementary material for: Genetic insights into dispersal distance and disperser fitness of African lions (Panthera leo) from the latitudinal extremes of the Kruger National Park, South Africa
Source: BMC Genet. 2018 Apr 3;19:21. doi: 10.1186/s12863-018-0607-x (PMC5883395; doi:10.1186/s12863-018-0607-x)
Supplement: Supplementary file 1 — Background information on the analysed lions. (DOCX 25 kb) [file 12863_2018_607_MOESM1_ESM.docx]

**Northern Kruger lions**

Pride G (Boyela pride)

Sampling date: 06-08-1999 - 13-10-2004

Centre of territory, coordinates: -22.837, 31.299

Territory size: 601km^2^

The Boyela Pride consisted of four adult males and ten females with three six month old cubs, three sub adult females and two sub adult males at the onset of monitoring. This can be considered to be the “super pride” of the North as all neighbouring prides are related to them. It continued to exist during the study period (1999-2004).

Individual IDs: 150.07, 150.11, 150.13, Km15 (150.05), Km17 (150.03), Kru03 (4327566122), Kru11 (150.09)

150.03 Last seen on the 3/08/2002 and near Mavatsane with one of the other 3 coalition members. This was on the periphery of their territory and they were associating with unknown unidentified lionesses. This coalition was probably ousted or left voluntarily as males 150.15 and 411576074A from Langtoon were associating with some of the Boyela females.

150.05 Was killed by buffaloes at Nwarihlangari windmill on the 16/07/2003. He was limping and in 3/5 condition and had been single for approximately three months. The carcass was too decomposed to collect sections for histology. However no visible clinical sign of tuberculosis could be found and sections collected had negative culture results.

150.07 Last seen on 18/07/2003, and could not be relocated in spite of numerous efforts of calling and monitoring her natal pride. Her radio collar was torn, suggesting a traumatic incident.

150.09 She was still living and in good condition when the study was terminated. She raised 3 cubs to adulthood during the study and two of these young females were recruited into their natal pride.

150.13 She lost condition and was euthanized on the 05/12/2002. Reasons for loss in condition could not be determined except that she may have been older than originally estimated. She was the largest female ever weighed in Kruger (182.5 kg). She also raised two female cubs and both were recruited into their natal pride.

Pride C (Nkulumbeni pride)

Sampling date: 07-08-1999 - 23-04-2002

Centre of territory, coordinates: -23.027, 31.365

Territory size: 288.8 km^2^

The Nkulumbeni Pride consisted of five adult males (apparent takeover and new and younger), eight females and two sub adult males. When this pride was selected and processed it appeared as though they were in a take-over process. The ousted coalition consisted of three males that were also associating with a female pride from the Langtoon Dam area at the time. This pride continued to exist during the study period (1999-2004).

Individual IDs: 434C467020, 150.25, Km10 (150.21), Km14 (150.23)

150.21 Part of the Initial Nkulumbeni South Coalition. He survived to the end of the study.

150.23 Part of the initial Nklumbeni South Coalition. During the last 6 months of the study he and 150.21 were ousted from the Boyela Pride by two previously identified peripheral males. 150.23 was paralyzed after he was bitten over the lumbo-sacral area. His spinal cord was fractured in the process and he was euthanized - the only Northern male that a complete necropsy could be done on.

150.25 A six-year-old female at the start of the study. She raised 4 cubs past one year of age at the end of the study. However she previously lost 6 cubs during the study. She survived until the study was terminated.

Pride K&F = Hlamalala/Elandskuil pride

Sampling date: 04-08-1999 – 25-04-2002

Centre of territory, coordinates: -22.665, 31.248

Territory size: 297.42 km^2^

The Hlamalala/Elandskuil Pride consisted of eight females. During the first three years unknown and unidentified adult males were seen from the air associating with them but at irregular intervals. In general only two territorial males could be associated with them during the five years. It could never be determined who sired their cubs. This pride lives in a territory that is being traversed frequently by Mozambican illegal immigrants. One of the females were killed with a bladed object while four others simply disappeared for no apparent reason until only one with three 18-month old sub adults were left.

Individual IDs: 150.01, 150.27, 150.29, 20PLCC, 20PLDD, 4115275A2B, 43271F4945, Km16 (4327354E6F)

150.01 Adult female killed with a bladed object through the scapula presumably by an undocumented Mozambican immigrant. Her carcass was discovered in the vicinity of the carcass of an adult elephant cow on 30/3/2004.

150.27 This six-year-old lioness left their territory for unknown reasons and was found on her own on the Shangoni section approximately 80 km south west of her core area. Her skull was picked up on 06/09/2000 in the remote area where she was last seen. The intact radio collar was also recovered.

150.29 She was the only surviving female from this pride and at the end managed to raise three cubs of which the two females stayed with her.

Pride U (Mpenza pride)

Sampling date: 11-10-2004

Centre of territory, -22.970, 31.438

Territory size: approximately 200 km^2^

This pride consisted of seven females. The five Nkulumbeni males also associated with them but it was difficult to determine if cubs were sired. They appeared to be related to the Boyela “super pride”.

Individual IDs: Kru06 (43471D1143), Kru08 (434B310863)

Pride I (Ndzophorini/Langtoon pride)

Sampling date: 24-04-2002

Centre of territory, coordinates: -22.837, 31.414

This pride consists of four adult females but only male samples were sent in because of the preponderance of females in the north of the Park. This apparent nomadic sub adult male coalition was still associating with their mothers while being sampled and were not evicted yet at the time.

Individual IDs: 4327082E21, Km06 (434D281B25), Km18 (434C5F223C)

Pride H (Biesiesvlei pride)

Sampling date: 11-10-2004

Centre of territory, coordinates: -23.099, 31.494

Territory size: approximately 150 km^2^

This is a relatively small pride that resides north of the eastern sector of the Shingwedzi River up to the Mozambican boundary.

Individual IDs: Kru04 (441567391C), Kru13 (442D285E7D), Kru14 (442E0A1B5E)

Pride Q (Phukwane pride)

Sampling date: 16-05-2000 – 18-05-2000

Centre of territory, coordinates: -22.989, 30.956

This was a pride of ten lions, but renowned for cattle killing. Members got killed by neighbouring communities at frequent intervals and temporary vacancies developed.

Individual IDs: SHPL01, SHPL02, SHPL03, SHPL04, SHPL05, SHPL12

Pride R (Shangoni Koppies pride)

Sampling date: 17-05-2000

Centre of territory, coordinates: -23.232, 31.002

This was quite a large pride of 22 individuals.

Individual IDs: SHPL06, SHPL08

Pride L (Magamba male coalition, non-territorial)

Sampling date: 25-04-2002

Sampling site, coordinates: -22.778, 31.191

This nomadic group of five males was first seen in April of 2002. They later migrated to another territory and settled as territorial males in 2004 (Malahlapanga: -22.877, 31.026).

Individual IDs: Km01 (434E504603), Km02 (434C69347C), Km05 (434C457170), Km08 (4324730B36), Km09 (4326311472)

Single Km04 (7F7D525419, Biesiesvlei)

Sampling date: 22-04-2002

Sampling site, coordinates: -23.073, 31.469

This male resided in the territory of Pride H during 2002. His origin was unknown.

Single Km07 (Minga, Punda Maria gate)

Sampling date: 09-12-1999

Sampling site, coordinates: -22.736, 30.984

Male shot outside Punda Maria gate after he had killed 28 head of cattle over a period of weeks. He was in prime condition and approximately six years old. He was never seen to associate with other lions while on his killing sprees.

Single Km13 (43274C0942, Babalala/Papanyane)

Sampling date: 29-04-2001

Sampling site, coordinates: -22.902, 31.283

An old but prime unknown male arriving at a calling station but never seen again subsequently.

Single Kru12 (432718572C, Langtoon)

Sampling date: 13-10-2004

Sample site: -22.823, 31.283

He was part of a two member coalition that took over the Boyela Pride during 2002/3. He was only sampled while on his own but was a well monitored lion. However his origin was unknown.

Single Kru 07 (4323583C3A, Nwaswitshumbe)

Sampling date: 13-10-2004

Sampling site, coordinates: -22.837, 31.299

Previously classified as a single but subsequently seen to be associating with Boyela females.

**Southern Kruger lions**

Pride A (new Crocodile Bridge pride)

Sampling date: 07-09-2004

Centre of territory, coordinates: -25.342, 31.888

Territory size: 41.12 km^2^

This was a new group of four unknown females that moved into a historically known and distinct territory. They were joined by two previously identified nomadic males that now became territorial. By July 2007 none of these lions could be found in this territory and a completely new pride of females with cubs were present. The previous known pride was gradually evicted by seven nomadic males.

Individual IDs: Kru28 (442E125740), Kru29 (4415580263), Kru31 (441F417D7E), Kru34 (434E747168), Kru41 (44156C4560), Kru47 (4326347624)

Pride B (Malelane pride)

Sampling date: 08-05-2000 – 27-09-2001

Centre of territory, coordinates: -25.432, 31.484

Territory size: approximately 60 km^2^

This pride consisted of six adult lionesses and two territorial males. They have already been a historically established pride and not newly formed.

Individual IDs: 879, 4326255837, 20PL02, 40600B411C, 43260D561F

Pride D (Nwatimhiri pride)

Sampling date: 22-05-2000

Centre of territory, coordinates: -25.108, 31.717

Territory size: unknown

This pride consisted of three females and two territorial males. They were an old established pride at the time of sampling.

Individual IDs: 414F503E2C

Pride E (Gomondwane pride)

Sampling date: 11-10-1999 – 08-09-2004

Centre of territory, coordinates: -25.254, 31.845

Territory size : 89.09 km^2^

The Gomondwane Pride consisted of two territorial males and eight females. Male 405D78504F was a new but elderly male associating with the females after the death of the two previous territorial males. However all three males were originally identified for the first time in a nomadic group during April of 1999. The Gomondwane pride remained remarkably intact and stable during the study period (1999-2004).

Individual IDs: Kru21 (4415710E6B), Kru30 (7F7D522643), Kru33 (7F7D3D2B0F), Kru36 (44155D142B), Kru43 (405D78504F), Kru44 (7F7D3D122B), 413875065065B, Km23 (405D6F281E)

Pride Ex-A (original Crocodile Bridge pride)

Sampling date: 18-06-1998 – 11-12-2001

Centre of territory, coordinates: -25.342, 31.888

Territory size: 41.12 km^2^

The original Crocodile Bridge Pride consisted of two territorial males and five females. This is the pride that was gradually evicted by the seven nomadic males. The nomadic males stayed in the territory for approximately three months during which the females with cubs attempted to avoid them while non-lactating females tolerated them. Ultimately all cubs disappeared and only two females remained – one emaciated and the other settling in neighbouring Nkongoma territory. Both were necropsied during the next six months and found to be suffering from advanced tuberculosis. The damaged and abandoned radio collar of one nomadic male was picked up in the Crocodile Bridge territory and the male was relocated again some months later in the neighbouring Nkongoma area where he eventually became emaciated and was euthanized.

Individual IDs: 40693E1116, 150.53, 20PL14, 20PLAA, Km24 (405E020D0E), TSB10977

Pride T (Mpondo pride)

Sampling date: 08-06-2001

Sampling site, coordinates: -25.213, 31.722

Territory size: 90 km^2^

This pride consisted of two females and two territorial males. This was an old established pride at the time of sampling.

Individual IDs: Km26 (4326343247), Km30 (4326360A1B)

Pride Hb (Sabie Park males, non-territorial)

Sampling date: 11-02-2001

Sampling site, coordinates: 24.980, 31.473

This group consisted of four sub adult to young adult males. They were captured as problem animals and returned to the Park.

Individual IDs: 432622700D, 432709281B, 432A7B682D, 4327397C7D

Pride J (Mtetamusha males, non-territorial)

Sampling date: 15-06-2001

Sampling site, coordinates: -25.447, 31.281

This group consisted of five adult males. They were captured as problem animals and returned to the Park.

Individual IDs: 7F7D0E5F2C, Km25 (7F7D3B1F44), Km28 (7F7D525C48), Km31 (7F7D4A574A)

Pride M (Mativuhlungu pride, non-territorial)

Sampling date: 09-09-2004 – 10-10-2004

Sampling site, coordinates: -25.149, 31.899

This is a complex group as possible territorial females (determined through age) were in the company of previously unidentified males and females. Sixteen different lions were sampled in this area of which four were females and twelve were males. None of these animals were found in this area after first time sampling but some were found again at various other locations in the South of Kruger.

Individual IDs: Kru15 (441F334C5D), Kru17 (456A7E3D00), Kru18 (456A550205), Kru26 (442D31795C), Kru38 (432640085E), Kru40 (434C470204), Kru42 (456D4F4562), Kru45 (456B176607)

Pride N (Powerlines pride, non-territorial)

Sampling date: 09-09-2004

Sampling site, coordinates: -25.218, 31.877

This is a complex group of apparent nomadic lions. Ten lions were sampled here but were never seen in the sampling area again but later at various other sites in different known territories.

Individual IDs: Kru16 (441F4A251B), Kru23 (434D0F5B34)

Pride O (Mpanamana pride, non-territorial)

Sampling date: 08-09-2004

Sampling site, coordinates: -25.301, 31.953

This is a nomadic group of lions. Seven males and two females were sampled.

Individual IDs: Kru19 (441F321408), Kru22 (441F3F260E), Kru25 (4415514E05), Kru27 (4415633833), Kru35 (441550741E), Kru39 (434C616E5E), Kru46 (434D092D75), Kru48 (434F1F6314), Kru49 (442C4D655B)

Pride P (Jeep Road pride)

Sampling date: 09-09-2004 – 10-09-2004

Sample site, coordinates: -25.176, 31.970

Eight lions were sampled of which two females were analysed. No visible pride structure was detectable amongst lions seen in this area even though they appeared to have accepted each other. None of them were found again during subsequent surveys.

Individual IDs: Kru24 (456A234500), Kru32 (456A6E4223)

Single 20PL21 (Muhlamamadube)

Sampling date: 26-10-2000

Sampling site, coordinates: -25.240, 31.563

She was a single emaciated lioness that could not be related to a pride with certainty but mainly because the prides in the area were not intensively monitored. She was positive for Feline Immunodeficiency Virus but negative for Bovine Tuberculosis.

Single 21PL10 (Lubye Lubye)

Sampling date: 11-05-2001

Sampling site, coordinates: 25.104, 31.883

A single old emaciated lioness with unknown pride history. She was positive for Feline Immunodeficiency Virus and Bovine Tuberculosis.

Single Km12 (41160B2F49, Delaport)

Sampling date: 22-02-2001

Sampling site, coordinates: -25.058, 31.568

Young nomadic male, very emaciated but negative for Feline Immunodeficiency Virus and Bovine Tuberculosis. Had intermittent company from other males but could not keep up with them.

Single Km19 (4326693031, Lower Sabie Road)

Sampling date: 15-01-2002

Sampling site, coordinates: -24.987, 31.632

Emaciated adult male of unknown origin. Positive for Feline Immunodeficiency Virus and Bovine Tuberculosis.

Single Km20 (20PL09, Nursery Road Skukuza)

20PL09 Nursery Road Skukuza

Sampling date: 20-03-2000

Sampling site, coordinates: -24.996, 31.561

An emaciated young male in a group of nomadic sub adults. Positive for Feline Immunodeficiency Virus and Bovine Tuberculosis.

Single Km29 (21PL17, Kruger Gate)

Sampling date: 28-08-2001

Sample site, coordinates: Lat: -24.9880545, Lon: 31.4921276

Emaciated young male severely bitten over the lumbo-sacral area. Positive for Feline Immunodeficiency Virus and Bovine Tuberculosis.

Single Kru20 (92830, Nhlanganzwane)

Sampling date: 09-09-2004

Sample site, coordinates: -25.244, 31.974

Part of a group of three nomadic lions (one male, two females) of unknown origin that evicted the established resident pride in this particular territory. They were lodging in this territory for approximately 18 months before they took over. She was seen in 2004 with a ten month old male cub – both in good condition and again in 2007.
