# Supplementary material for: Genetic insights into dispersal distance and disperser fitness of African lions (Panthera leo) from the latitudinal extremes of the Kruger National Park, South Africa
Source: BMC Genet. 2018 Apr 3;19:21. doi: 10.1186/s12863-018-0607-x (PMC5883395; doi:10.1186/s12863-018-0607-x)
Supplement: Supplementary file 3 — Map of sampling localities. (DOCX 793 kb) [file 12863_2018_607_MOESM3_ESM.docx]

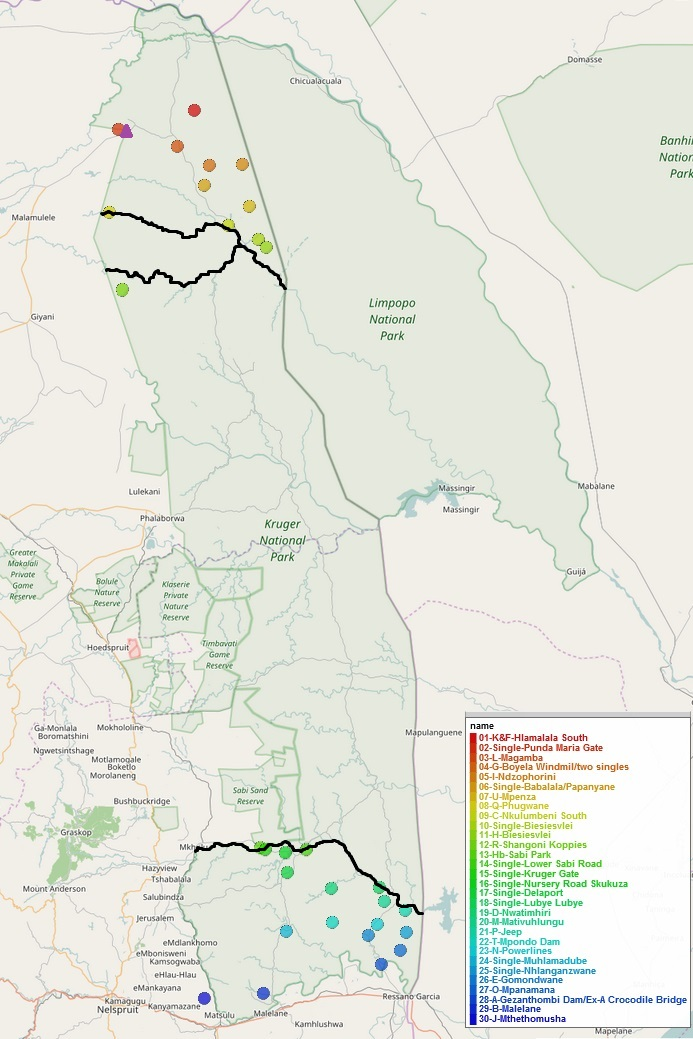


**Map of sampling localities.**

Purple triangle: Punda Maria gate, black lines (north to south): Phugwane, Shingwedzi and Sabie Rivers. © OpenStreetMap contributors. The data is available under the Open Database License, and the cartography is licensed as CC BY-SA.
