# Supplementary material for: Genetic insights into dispersal distance and disperser fitness of African lions (Panthera leo) from the latitudinal extremes of the Kruger National Park, South Africa
Source: BMC Genet. 2018 Apr 3;19:21. doi: 10.1186/s12863-018-0607-x (PMC5883395; doi:10.1186/s12863-018-0607-x)
Supplement: Supplementary file 4 — Table with some characteristics of the northern and southern subpopulation. (DOCX 20 kb) [file 12863_2018_607_MOESM4_ESM.docx]

**Some characteristics of the northern and southern subpopulation (1998-2004).**

| Parameter | Northern Kruger | Southern Kruger |
| --- | --- | --- |
| Lion density (ind./100km^2^)^1^ | 2.7-3.1 | 7.0-8.1 |
| Adult sex ratio (♀:♂)^1^ | 1.75:1 | 1.06:1 |
| Female territory size (km^2^)^2^ | 300 (range 150-600) | 70 (range 40-90) |
| Body condition  (percentage with BCS=5)^3^ | 77% (95% CI: 63-87%) | 44% (95% CI: 32-56%) |
| BTB prevalence | not observed (44 lions [1], 20 lions in this study) | 72% (95% CI: 64-80%) [2] |
| FIV prevalence | 58% (95% CI: 50-65%) [2] | 63% (95% CI: 57-68%) [2] |

1: Based on three surveys with all-night calling stations during the study period at which 76 lions were observed in northern Kruger and 98 lions in southern Kruger.

2. Prides were defined when groups arrived simultaneously at calling stations and when acceptance and affection was shown to latecomers. Frequency of subsequent associations was also used as an indicator of pride relation status. Territory sizes from five northern and five southern prides were estimated using 95% minimum convex polygon based on radio-telemetry data collected during the study period, opportunistic observations and annual lion surveys. VHS Radio transmitters were fitted to the necks of 22 adult females from six prides (three in the north and three in the south), which were radio-tracked every three months from a helicopter. When a lion had lost condition to the extent that it had reached a 3/5 score rating, the animal was tracked once a month from the air as well as from the ground. Once it deteriorated to 2/5 condition it was tracked weekly. GIS mapping was based on aerial and ground radio tracking analysis (ArcGIS, ArcView from ESRI version 9.2).

3. Body condition score (BCS) was assessed on a scale from 1 (very poor) to 5 (excellent) [2].

**References**

1. Keet DF, Michel AL, Bengis RG, Becker P, van Dyk DS, van Vuuren M, Rutten V, Penzhorn BL: **Intradermal tuberculin testing of wild African lions (*Panthera leo*) naturally exposed to infection with Mycobacterium bovis**. *Vet Microbiol* 2010, **144**(3-4):384-391.

2. Maas M, Keet DF, Rutten V, Heesterbeek JAP, Nielen M: **Assessing the impact of feline immunodeficiency virus and bovine tuberculosis co-infection in African lions**. *Proc R Soc B-Biol Sci* 2012, **279**(1745):4206-4214.
