# Supplementary material for: Genetic insights into dispersal distance and disperser fitness of African lions (Panthera leo) from the latitudinal extremes of the Kruger National Park, South Africa
Source: BMC Genet. 2018 Apr 3;19:21. doi: 10.1186/s12863-018-0607-x (PMC5883395; doi:10.1186/s12863-018-0607-x)
Supplement: Supplementary file 5 — No discernible effect of lion age and sampling date on isolation-by-distance analyses. (DOCX 12 kb) [file 12863_2018_607_MOESM5_ESM.docx]

**No discernible effect of lion age and sampling date on isolation-by-distance analyses.**

As patterns of isolation-by-distance may have been influenced by the fact that samples were collected in different years and that the sampled individuals were of different age, the influence of differences in age and collection year was analysed by traditional multiple regressions per sex and per region, with *r*_LR_ as dependent variable and geographical distance (km) and various time-age related parameters per pair of individuals as independent variables (difference in sampling date, difference in birth date, difference in age at the moment sampling, difference in age (difference in age at the moment of sampling minus difference in birth date), average age). This procedure overestimated significance due to the wrongful assumption of independence of pairwise data. Despite this overestimation, only in one instance was significance for another variable other than geographical distance observed; namely difference in birth date among males from northern Kruger. This variable was also significant when tested with the Mantel procedure whereby birth date was randomized among individuals (*P* = 0.03, *n*_individuals_ = 21). The correlation between *r*_LR_ and difference in birth date was positive, which may be attributed to an increase in the fraction of father-son pairs when animals were born years apart. However, there was no significance (*P* > 0.4) when potential daughters were also included (by including opposite-sex pairs of which the most recent born individual was a female). This suggests a spurious correlation instead, especially as parent-daughter pairs were more likely to be sampled than parent-son pairs considering the occurrence of male-biased dispersal in lions (also observed in this study, see Results section).
