# Supplementary material for: Genetic insights into dispersal distance and disperser fitness of African lions (Panthera leo) from the latitudinal extremes of the Kruger National Park, South Africa
Source: BMC Genet. 2018 Apr 3;19:21. doi: 10.1186/s12863-018-0607-x (PMC5883395; doi:10.1186/s12863-018-0607-x)
Supplement: Supplementary file 6 — Figure showing absence of isolation-by-distance among males ≥3 year old. (DOCX 98 kb) [file 12863_2018_607_MOESM6_ESM.docx]

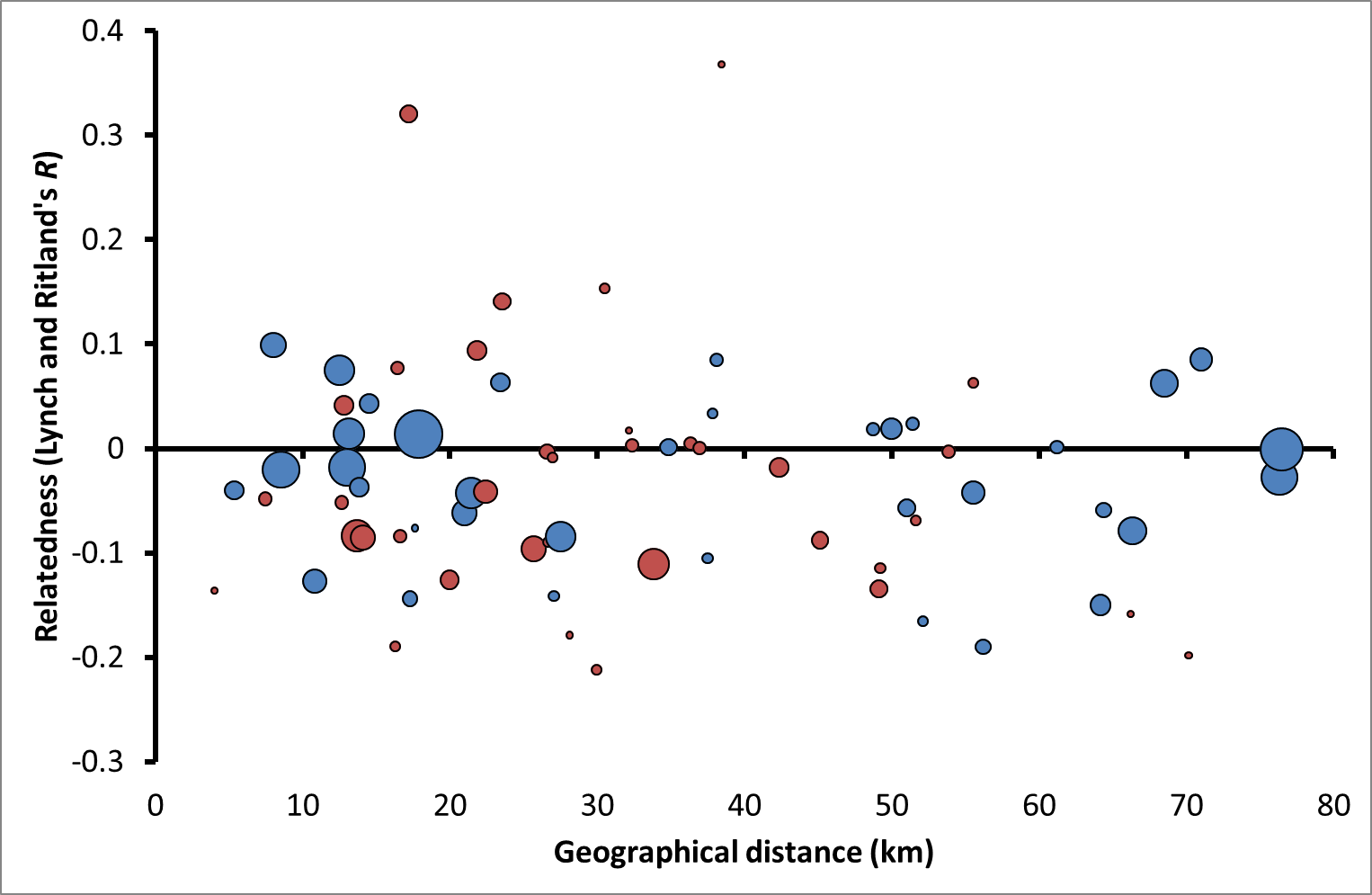


**Absence of isolation-by-distance among males ≥ 3 year old.**

Red circles: northern Kruger, blue circles: southern Kruger. Circles represent mean relatedness per pair of localities and circle size total number of comparisons per pair of localities (minimum = 1, maximum = 35).

Northern Kruger: Pearson *r* = -0.08, *P* = 0.30, *n*_individuals_ = 19, *n*_localities_ = 9; southern Kruger: Pearson *r* = -0.03, *P* = 0.32, *n*_individuals_ = 28, *n*_localities_ = 9.
