# Supplementary material for: Genetic insights into dispersal distance and disperser fitness of African lions (Panthera leo) from the latitudinal extremes of the Kruger National Park, South Africa
Source: BMC Genet. 2018 Apr 3;19:21. doi: 10.1186/s12863-018-0607-x (PMC5883395; doi:10.1186/s12863-018-0607-x)
Supplement: Supplementary file 7 — Figure showing the number of microsatellite clusters based on the software Structure using the method of Evanno et al... 2005. (DOCX 52 kb) [file 12863_2018_607_MOESM7_ESM.docx]

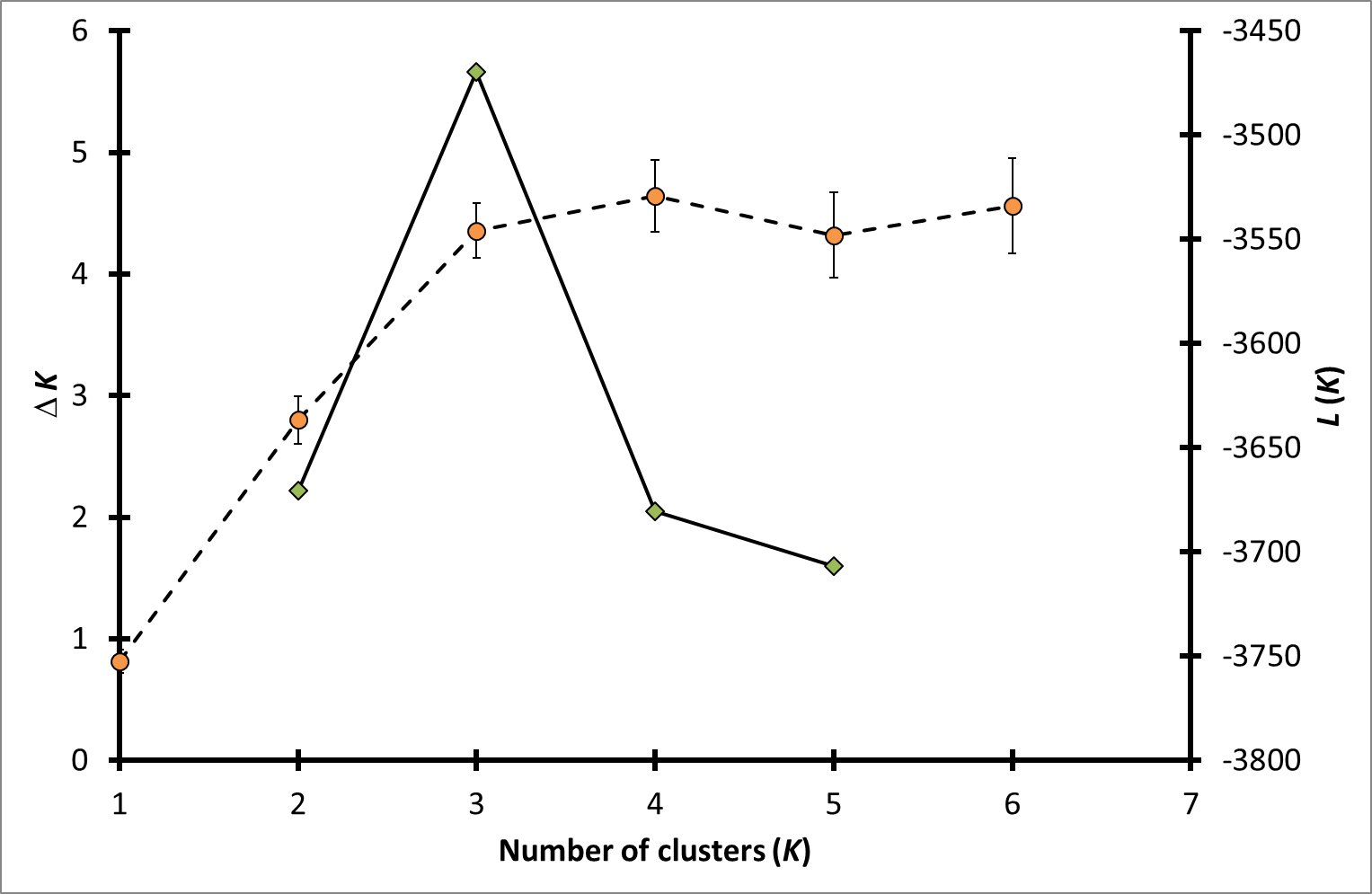


**Number of microsatellite clusters based on the software Structure using the method of Evanno et al. 2005 [1].**

Orange circles: mean *L*(*K*) (± SD) over 10 runs for each *K* value, green diamonds: *∆K*. The peak of *∆K* denotes the most likely number of clusters, here three.

**References**

1. Evanno G, Regnaut S, Goudet J: **Detecting the number of clusters of individuals using the software STRUCTURE: a simulation study**. *Mol Ecol* 2005, **14**(8):2611-2620.
