# Supplementary material for: Genetic insights into dispersal distance and disperser fitness of African lions (Panthera leo) from the latitudinal extremes of the Kruger National Park, South Africa
Source: BMC Genet. 2018 Apr 3;19:21. doi: 10.1186/s12863-018-0607-x (PMC5883395; doi:10.1186/s12863-018-0607-x)
Supplement: Supplementary file 8 — Figure showing the fraction of candidate residents, candidate dispersers and individuals with mixed ancestry per locality. (DOCX 37 kb) [file 12863_2018_607_MOESM8_ESM.docx]

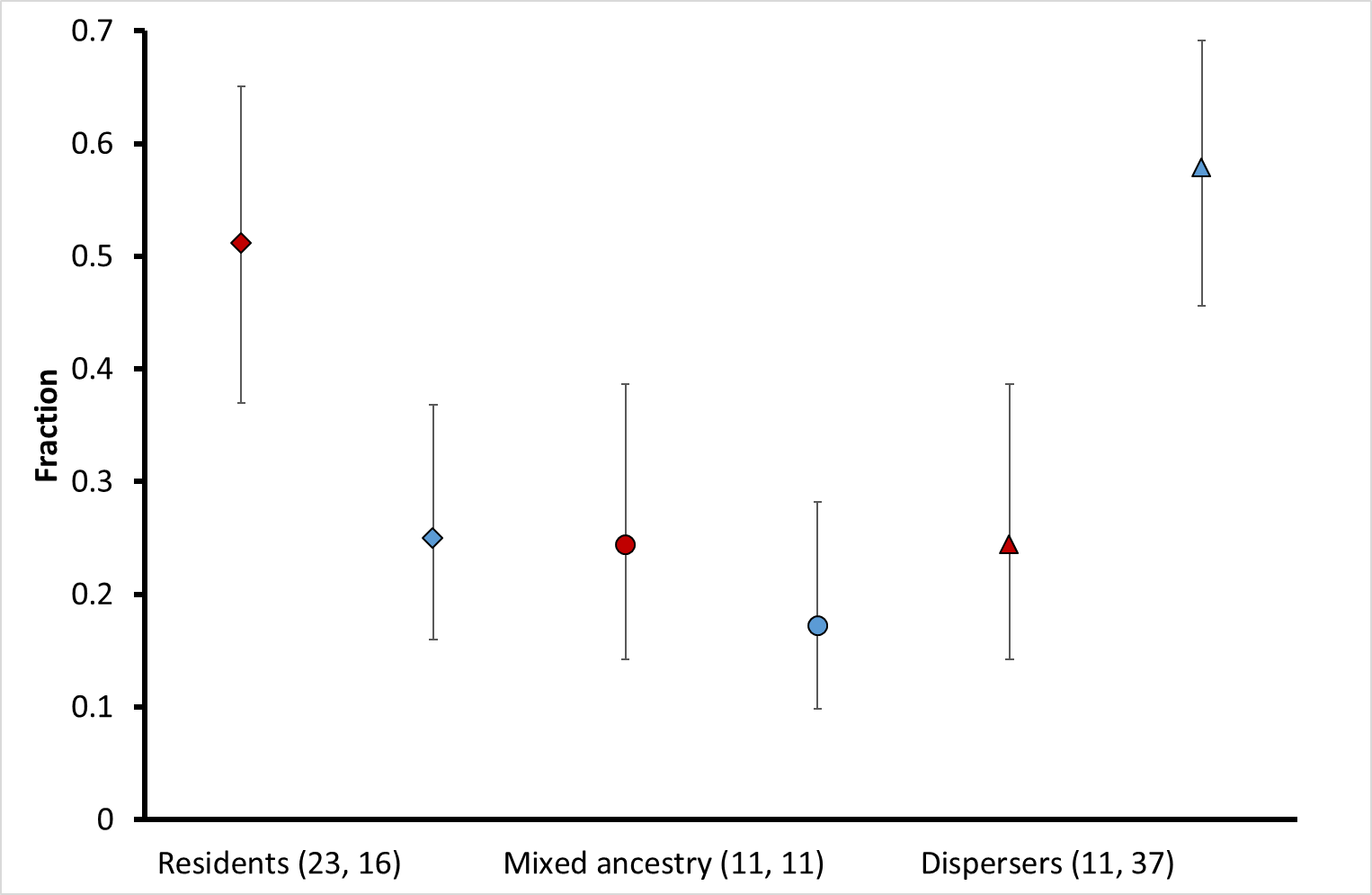


**Fraction of candidate residents, candidate dispersers and individuals with mixed ancestry.**

Red data points: northern Kruger, blue data points: southern Kruger, diamonds: candidate residents, circles: individuals with mixed ancestry (probably consisting of both residents and dispersers), triangles: candidate dispersers, error bars: 95% confidence interval. Horizontal axis, values between brackets: sample size of respectively northern and southern Kruger.

Candidate resident: > 90% of the genetic make-up assigned to local microsatellite cluster, candidate disperser: < 20% of the genetic make-up assigned to local microsatellite cluster. Mixed ancestry: between 20% and 90% of the genetic make-up assigned to local microsatellite cluster.
