# Supplementary material for: Genetic insights into dispersal distance and disperser fitness of African lions (Panthera leo) from the latitudinal extremes of the Kruger National Park, South Africa
Source: BMC Genet. 2018 Apr 3;19:21. doi: 10.1186/s12863-018-0607-x (PMC5883395; doi:10.1186/s12863-018-0607-x)
Supplement: Supplementary file 9 — Figure showing the cumulative frequency distribution of the proportion of DNA per individual assigned to the local microsatellite cluster by the software Structure. (DOCX 56 kb) [file 12863_2018_607_MOESM9_ESM.docx]

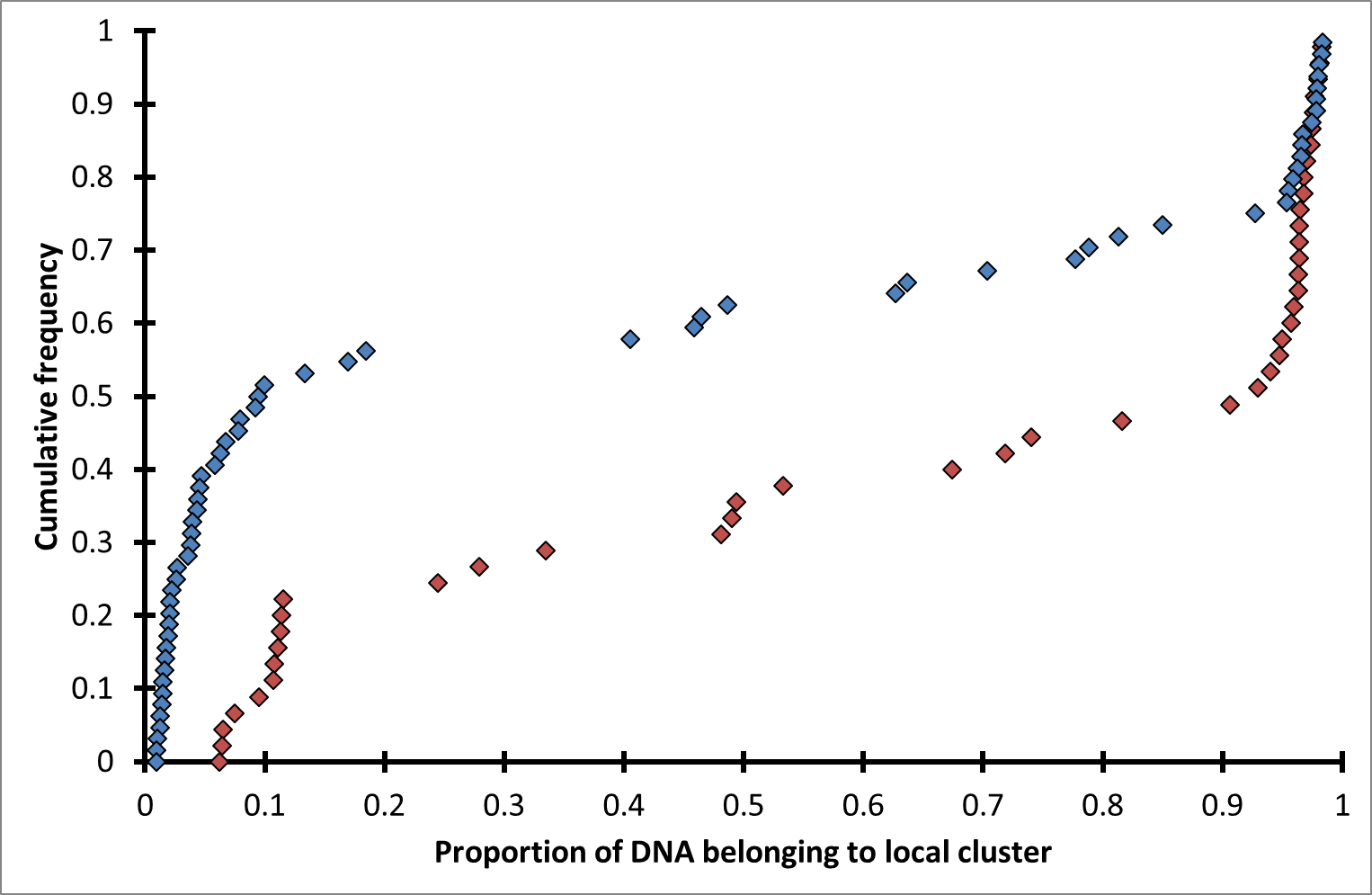


**Cumulative frequency distribution of the proportion of DNA per individual assigned to the local microsatellite cluster by the software Structure.**

Red data points: northern Kruger, blue data points: southern Kruger.
