# Supplementary material for: Genetic insights into dispersal distance and disperser fitness of African lions (Panthera leo) from the latitudinal extremes of the Kruger National Park, South Africa
Source: BMC Genet. 2018 Apr 3;19:21. doi: 10.1186/s12863-018-0607-x (PMC5883395; doi:10.1186/s12863-018-0607-x)
Supplement: Supplementary file 10 — Figure showing the relationship between body condition and age per locality (logistic regression). (DOCX 72 kb) [file 12863_2018_607_MOESM10_ESM.docx]

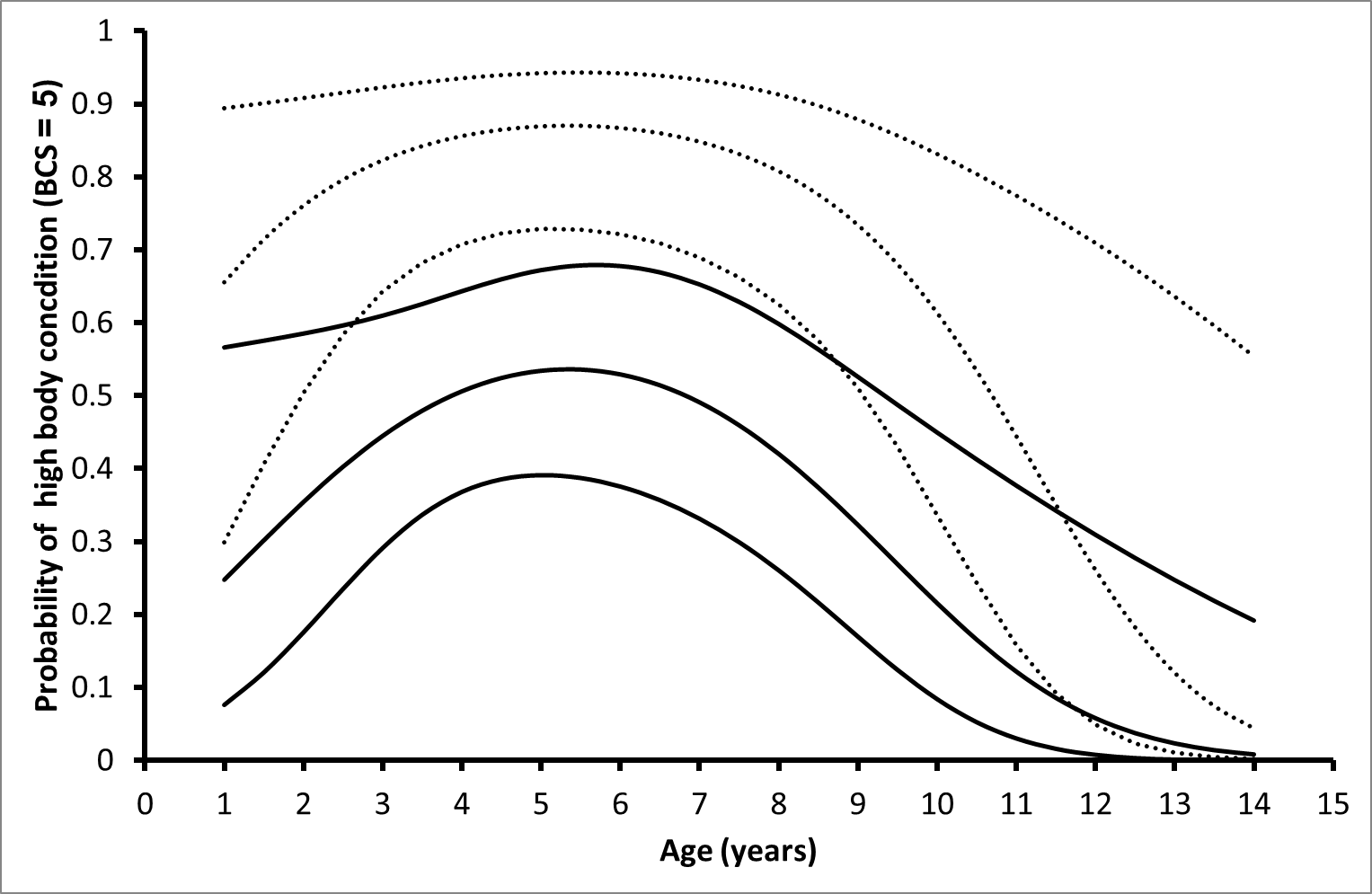


**Relationship between body condition and age per locality (logistic regression).** Regression lines: probability of having a high body condition (BCS = 5), upper and lower lines: upper and lower limit of the 95% confidence interval of the regression lines, dotted lines: northern Kruger, solid lines: southern Kruger.

Dependent variable: body condition (BCS): 0: BCS ≤ 4, 1: BCS = 5. *χ*^2^-value model = 24.47, d.f. = 3, *P*_model_ < 0.0001, *P*_age_ = 0.037, *P*_age_^2^ = 0.014, *P*_north-south_ = 0.00039 (higher body condition in the north). Sex was not significant when added to the regression model (*P*_sex_ = 0.12). Northern individual with body condition = 0: *n*_individuals_ = 10, northern individual with body condition = 1: *n*_individuals_ = 34, southern individual with body condition = 0: *n*_individuals_ = 35, southern individual with body condition = 1: *n*_individuals_ = 27.
